# A simple and powerful test of vaccine waning

**DOI:** 10.1093/aje/kwag118

**Published:** 2026-07-01

**Authors:** Gellért Perényi, Matias Janvin, Mats J Stensrud

**Affiliations:** Institute of Mathematics, École Polytechnique Fédérale de Lausanne (EPFL), Rte Cantonale, 1015, Lausanne, Switzerland; Oslo Centre for Biostatistics and Epidemiology, Department of Biostatistics, University of Oslo, 0372 Oslo, Norway; Department of Surgery and Anesthesiology, Diakonhjemmet Hospital, Oslo, Norway; Institute of Mathematics, École Polytechnique Fédérale de Lausanne (EPFL), Rte Cantonale, 1015, Lausanne, Switzerland

**Keywords:** causal inference, challenge trial, hypothesis test, vaccine waning

## Abstract

Determining whether vaccine efficacy wanes is important for individual and public decision making. Yet, quantification of waning is a subtle task. The classical approaches cannot be interpreted as measures of declining efficacy unless we impose unreasonable assumptions. Recently, formal causal estimands designed to quantify vaccine waning have been proposed. These estimands can be bounded under weaker assumptions, but the bounds are often too wide to make claims about the presence of waning. We propose a different approach: a formal test to assess whether a treatment effect is constant over time at the individual level. This test provides a considerable power gain over existing approaches and is valid under interpretable assumptions in vaccine trials. We illustrate the increase in power through real and simulated examples, using 3 different approaches to compute the test statistics. Two of these approaches are based solely on summary data, accessible from existing clinical trials. Beyond our test, we also give new results that bound the waning effect. We use our methods to reanalyze data from a randomized controlled trial of the BNT162b2 COVID-19 vaccine. While a prior analysis did not establish waning, our test rejects the null hypothesis of no waning.

## Introduction

**Figure 1.**
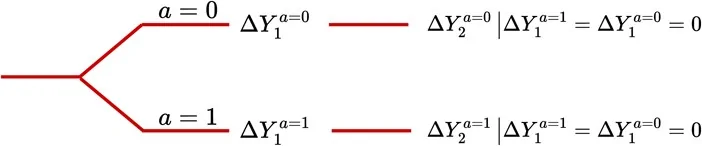
Illustration of outcomes in a 2-arm randomized controlled trial over 2 intervals. Second interval observation is conditional on being event-free at time 1.

Consider a randomized controlled trial (RCT) evaluating a vaccine against an infectious disease (eg, HIV). Using data from the RCT, researchers estimated vaccine efficacy (VE) to be *0.8* one month after vaccination. To assess if the VE changed at later times, the participants who were event-free in the first analysis were included in a subsequent analysis at 3 months that gave a $\widehat{VE}=0.6$ (see Figure 1). The researchers concluded that the efficacy had decreased at 3 months; that is, the vaccine effect had waned.

A key question is whether such a contrast of VEs at different times gives a meaningful notion of waning. To answer this question, we take for granted that the term waning alludes to a declining causal effect; that is, vaccine protection that decreases over time. To draw a valid inference on a causal effect, the populations being compared under vaccination or no vaccination (eg, placebo) should be exchangeable. Conceptually, this means that the distributions of risk factors for infection are expected to be similar in the two treatment groups (see, eg, [9]). Randomization ensures exchangeability for the comparison at the time of the first analysis (at 1 month). By the time of the subsequent analysis (at 3 months), however, conditioning on being event-free induces postrandomization selection: the participants who were event-free under vaccination are likely to differ from those who were event-free without vaccination. There is an additional problem when comparing VEs over time. Even within the same treatment arms, the analyses at the two subsequent intervals are estimated in different populations, making the declining VEs hard to interpret. See Figure 3 for the changing distribution of susceptibility over time and across treatment arms.

These concerns are not merely theoretical; heterogeneity in susceptibility is widespread. To fix ideas, we give an extreme example. Consider a trial of a vaccine against CCR5-tropic HIV^15^. Suppose that all individuals were in contact with the infectious agent, that is, they were naturally “challenged,” within the first month. The VE was estimated to be *0.8* after 1 month. Suppose further that the individual-level effect of the vaccine is constant in time: more precisely, had we challenged individuals with an infectious agent immediately after a 1-month isolation period, we would have exactly the same outcome as if we had challenged the individuals immediately after a 3-month isolation period. Then, the untreated participants who were not infected after the challenge must be inherently immune to the disease (eg, homozygous for the CCR5 *Δ 32* gene variant). Thus, by the time of the second analysis, all unprotected trial participants, regardless of the arm, have already experienced the outcome, while those who are protected, either by the vaccine or their genetically induced immunity, will remain event-free at time 2, either by the lack of exposure or by their immunity. It follows that no new events will occur in either of the 2 arms, and the conventional VE estimate would be equal to *0*. This estimate, however, contradicts the fact that the vaccine effect, on an individual level, is time-invariant.

This extreme example illustrates a point: conventional VE estimates cannot be used to establish waning. Such analyses are subject to selection bias due to depletion of susceptibles, corresponding to well-known issues with the interpretation of hazard ratios (HRs; see, eg, [3, 10]). In practice, the magnitude of bias is driven by unknown heterogeneity in the outcome risk and can, in principle, be large, although likely not as extreme as in our illustrative example. However, we can quantify the difference between conventional waning estimands and causal waning estimands under given levels of heterogeneity (see Appendix S1 for details).

Recognizing the problems with the conventional waning analyses, Janvin and Stensrud [12] proposed an alternative estimand for VE, based on the idea of challenge trials, building on the work of Stensrud and Smith [21]. In particular, their estimand could be computed in a hypothetical target trial with controlled exposure of the participants [2, 19]. Such trials are rarely conducted, as exposing participants, regardless of their health status, to infectious pathogens is unethical [11]. Vaccinating a (random) subset of participants before pathogen emergence is sometimes a compelling alternative to a challenge trial [13, 16], but requires knowledge of the timing of the infectious and noninfectious periods. While this design does not permit point identification of the challenge effects without additional assumptions, it can detect waning under mild assumptions. Furthermore, with data from conventional RCTs, the waning estimand of interest is only partially identifiable, that is, it is bounded under reasonable assumptions. Here, we introduce another approach to assess the existence of vaccine waning. Our work is grounded in a formal causal formulation of a declining treatment effect, related to Janvin and Stensrud [12]. However, unlike Janvin and Stensrud [12], we formulate a sharp null hypothesis of no waning and propose a test that is powerful, simple, and assumption-lean. In particular, the test can be conducted under reasonable assumptions in conventional vaccine trials, either using individual-level or summary data. We furthermore suggest a strategy in Appendix S2.1, based on some additional in-principle testable assumptions, to improve the bounds derived by Janvin and Stensrud [12].

## Observed data structure

Consider i.i.d. data from an RCT in which individuals are assigned to treatment $A \in \{0,1\}$ corresponding to placebo or vaccine. Define $Y_{k} \in \{0,1\}$ as an indicator of at least one outcome occurring by the end of interval *k*. Therefore, $Y_{k}\geq Y_{k^{\prime}}$ for all $k>k^{\prime}$. Then, $\Delta Y_{k}:= Y_{k}-Y_{k-1}$ is an indicator of whether the first outcome has occurred in interval *k*. To simplify the presentation, we will treat these intervals as discrete. Yet, the test is valid in a continuous-time setting as well, with minor modifications. As the data are assumed to be i.i.d., the subscripts indicating individuals are omitted. The notation in Figure 1 stands for potential outcomes under different levels of treatment. For example, $\Delta Y_{1}^{a=0}$ is the outcome at time 1 under an intervention that sets the treatment to *a=0*.

As highlighted in Section 1, the VE at time 2 is not a causal contrast. Consider instead a contrast among those who would survive the first interval, regardless of the treatment assignment, 


\begin{align*} &E[\Delta Y_{2}^{a=1}| \Delta Y_{1}^{a=1}=\Delta Y_{1}^{a=0}=0] \quad \textrm{vs} \\ &E[\Delta Y_{2}^{a=0}| \Delta Y_{1}^{a=1}=\Delta Y_{1}^{a=0}=0]. \end{align*}

By restricting the analysis to those who survive under both treatment assignments, the two populations are comparable at time 2. This contrast is an example of principal stratum effects (PSEs) [7].

However, we need to be careful when comparing effect contrasts among those who would survive under both levels of treatment at the second time to the same contrast among the general population at the first time: even if the analysis at each time has a causal interpretation, the two comparisons are computed in different populations. This complication concerning the PSE is particular to our setting and goes beyond the well-known critique of the PSE being a cross-world estimand [17, 18, 20].

**Figure 2.**
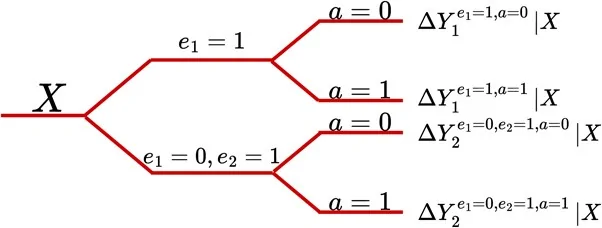
Four-arm randomized controlled trial: Individuals are randomized to treatment and to the time of the exposure. Due to isolation, those who are assigned to time 2 exposure are exchangeable with those who are assigned to exposure at time 1. *X* denotes the distribution of the baseline characteristics in the trial population, which is, by randomization and Assumption 1.1, expected to be identical in all 4 arms.

To illustrate our target estimand, consider instead an unconventional randomized 4-arm trial where participants are randomly assigned to one of the two levels of treatment and one of the two possible times of exposure (visualized in Figure 2).

Define exposure at time *k* as $E_{k} \in \{0,1\}$ for $k\in \{1,2\}$, corresponding to isolation ($E_{k}=0$), or exposure through some controlled procedure ($E_{k}=1$). We will not presume that we have data from this unconventional trial, but instead use this as a target trial, motivating our estimand.

In our derivations, we will use the following assumption from Stensrud and Smith [21]:

Assumption 1.1. (Exposure necessity).

\begin{align*} & E_k^a =0 \implies \Delta Y_k^{a}=0; \; E_2^{a, e_1=0}=0 \implies \Delta Y_2^{a, e_1=0} =0, \end{align*}

for all $a \in \{0,1\}, k \in \{1,2\}.$

The intervention on exposure happens at two times in the 4-arm trial. The potential outcome at time 1 is defined under the intervention setting exposure to 1, and the potential outcome at time 2 is defined under an intervention setting exposure at time 1 to be 0 and exposure at time 2 to be 1. Thus, in this hypothetical trial, participants are exposed to the pathogen according to a controlled procedure at a specific time.

**Figure 3.**
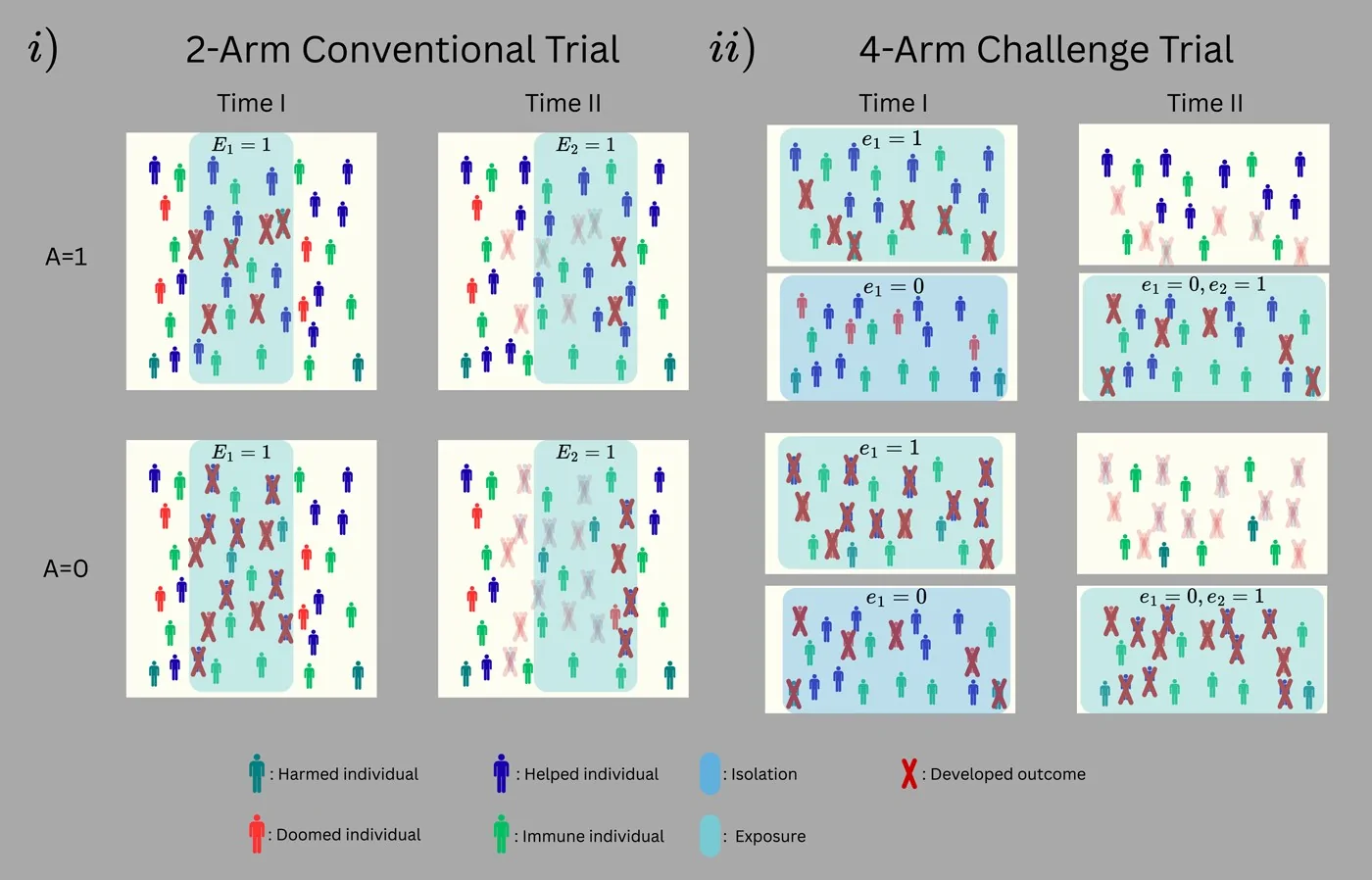
Illustration of two different trial designs. In conventional trials, subfigure (i), the populations under treatment and no treatment are exchangeable at baseline, without intervening on the exposure. Among exposed at time 1, the treated and the untreated groups are exchangeable as well, assuming treatment blinding, yielding an observed vaccine efficacy (VE) of *0.5* in this example. However, by time 2, the depletion of susceptibles (ie, selective exclusion from the at-risk population of the doomed and harmed or doomed and helped strata, depending on the assigned treatment), alters the distribution of susceptibility of the at-risk population. Even without waning, as the number of susceptibles declines more rapidly among the untreated, the observed vaccine efficacy declines to approximately *0.41*. In contrast, in a challenge trial, subfigure (ii), the 4 arms are exchangeable at baseline. Isolation ensures participants assigned to exposure at time 2 remain at risk until then, making them exchangeable at the time of the exposure as well. Consequently, the challenge vaccine efficacy is *0.5* at both times, equal to the proportion in the doomed-or-harmed populations divided by the proportion in the doomed-or-helped populations in this hypothetical example.

The potential outcome at time 2 is defined under an intervention that isolates individuals at time 1. Thus, those who were assigned to $e_{1}=0, e_{2}=1$ will not develop the outcome at the first time; they cannot be infected without exposure, following Assumption 1.1. The random assignment of treatment and exposure implies that the populations in all 4 arms are exchangeable (see Figure 3 for an illustration).

As proposed by Janvin and Stensrud [12], we define the challenge effects as follows:

Definition 1.2. (Marginal challenge effect).

(1) \begin{align*} VE_{1}^{\textrm{challenge}} &= 1- \frac{E[\Delta Y_{1}^{a=1, e_{1}=1}]}{E[\Delta Y_{1}^{a=0, e_{1}=1}]},\end{align*}

 

(2) \begin{align*} VE_{2}^{\textrm{challenge}} &= 1- \frac{E[\Delta Y_{2}^{a=1, e_{1}=0, e_{2}=1}]}{E[\Delta Y_{2}^{a=0, e_{1}=0, e_{2}=1}]} \end{align*}

The challenge effect conditional on baseline covariates is defined analogously (see Appendix S3 for details).

The challenge effect quantifies the effect of the vaccine in each interval in a way that is insensitive to the observed exposure pattern and the changing distribution of the unobserved heterogeneities over time. If $VE_{1}^{\textrm{challenge}}$ exceeds $VE_{2}^{\textrm{challenge}}$, then the protective effect of the vaccine has decreased over time, within the population that has been defined prerandomization [12].

While a “challenge trial,” such as the one illustrated in Figure 2, is sufficient to identify both $VE_{1}^{\textrm{challenge}}$ and $VE_{2}^{\textrm{challenge}}$, conducting such a trial is usually unethical, as deliberately exposing individuals to the pathogen could cause severe harm; hence, they are rarely conducted. Challenge trials can be run in healthy volunteers to minimize the risk of severe adverse effects; however, this approach typically fails to reflect the population of interest.

Janvin and Stensrud [12] derived conditions under which a waning parameter can be partially identified, even if the data are from a standard 2-arm RCT, in which exposure was neither intervened on nor measured, such as the one illustrated in Figure 1. Yet, the bounds derived for the second interval challenge effect can be wide, often including the first interval challenge effect, which prohibits a firm claim about the waning of the vaccine.

## A formal test

Consider the following sharp null hypothesis of no waning:

Hypothesis 1.3. (Sharp null hypothesis).

\begin{align*} & \Delta Y_1^{a, e_1=1} = \Delta Y_2^{a, e_1=0, e_2=1} \; w.p.1 \; \forall a \in \{0,1\}. \end{align*}

The sharp null hypothesis (1.3) states that anyone who would experience the outcome under exposure would do so regardless of whether exposure occurs at time 1 or at time 2. In short, individual susceptibility to exposure is constant across intervals and independent of exposure timing. One interpretation of Hypothesis 1.3 is that the vaccine has an all-or-nothing effect [8]; that is, under exposure and a given level of treatment, the potential outcomes are deterministic. This test might reject the null hypothesis of no sharp waning when potential outcomes are sensitive to exposure intensities, which can arise in settings with multiple strains circulating in the population. Thus, we also introduce an alternative test, which allows the exposure to take a continuous value, building on Janvin and Stensrud [12] (see Appendix S3 for details). However, this test often has substantially lower power.

We further restrict the exposure intervention to have no carry-over effect on the outcome. That is, exposure at time 1 cannot affect the outcome at time 2 except through survival. This restriction is compatible with a fixed incubation period of an infectious agent.

Assumption 1.4. (Exposure exclusion restriction).

\begin{align*} &\Delta Y_2^{a, e_1, \delta y_1=0, e_2=1}=\Delta Y_2^{a, \delta y_1=0, e_2=1} \quad \forall a, e_1 \in \{0,1\}^2\end{align*}

Assumption 1.4 holds if all causal paths from $E_{1}$ to $\Delta Y_{2}$ are intersected by $\Delta Y_{1}$ or $E_{2}$. However, it fails if exposure followed by survival at time 1 creates sustained immunity that prevents the development of the outcome at time 2, which can occur, for example, if asymptomatic infections are misclassified as no infections.

Remark 1. (Null hypothesis under Assumption 1.4).Suppose that Assumptions 1.1, 1.4, and 1.6 hold. An alternative formulation of the effect restriction using nested counterfactuals is

\begin{align*} & \Delta Y_2^{a, e_1, e_2=1}= \Delta Y_2^{a, e_1, \Delta Y_1^{a, e_1}, e_2=1}. \end{align*}

By Assumption 1.1 and 1.6, $\Delta Y_{1}^{a, e_{1}=0}=0$, thus

\begin{align*} &\Delta Y_{2}^{a, e_{1}=0, e_{2}=1}= \Delta Y_{2}^{a, e_{1}=0, \Delta Y_{1}^{a, e_{1}=0}, e_{2}=1}\\ &=\Delta Y_{2}^{a, e_{1}=0, \delta y_{1}=0, e_{2}=1} = \Delta Y_{2}^{a, e_{1}=1, \delta y_{1}=0, e_{2}=1}. \end{align*}

Therefore, under Assumptions 1.1 and 1.4, Hypothesis 1.3 is equivalent to  

\begin{align*} &\Delta Y_{1}^{a, e_{1}=1} \overset{H_{0}}{=} \Delta Y_{2}^{a, e_{1}=0, e_{2}=1} \\ &=\Delta Y_{2}^{a, e_{1}=1, \delta y_{1}=0, e_{2}=1}=\Delta Y_{2}^{a, e_{1}=0, \delta y_{1}=0, e_{2}=1}. \end{align*}

Suppose Hypothesis 1.3 holds. Consider an individual who was exposed in the first interval but remained event-free. This individual will remain event-free at time 2 as well, regardless of the time 2 exposure. This fact is related to a constraint on the distribution of joint counterfactuals under Hypothesis 1.3, which we discuss in Appendix S4.

Remark 2. (Population- vs individual-level waning).In principle, the challenge effects, given in Equations (1) and (2), can be equal at times 1 and 2 even if $\Delta Y_{1}^{a, e_{1}=1}\neq \Delta Y_{2}^{a, e_{1}=0, e_{2}=1} w.p>0$. However, assume that

\begin{align*} &\Delta Y_1^{a=0, e_1=1} = \Delta Y_2^{a=0, e_1=0, e_2=1} \; w.p.1, \end{align*}

but not necessarily for *a=1*. Conceptually, this assumption states that the risk of infection under placebo does not depend on the time of exposure. An argument supporting this assumption is that no active ingredient is administered in the placebo arm, and thus, the immune system’s response to exposure should not depend on the time to first exposure. Then, the population-level notion of no vaccine waning is equivalent to the sharp null Hypothesis 1.3, if there is no perfect cancellation in the following sense: exactly the same number of people experience “positive waning,” that is, the improvement of the vaccine over time, as the number of people who experience “negative waning,” that is, the deterioration of the vaccine over time. Such a perfect cancellation of positive and negative effects is unlikely. Thus, we can often intuitively treat the sharp null of no waning, and population-level no waning ($VE_{1}^{\mathrm{challenge}}=VE_{2}^{\textrm{challenge}})$ as equivalent. We provide a more formal argument building on the principal strata framework in Appendix S4.

The next results will lead to a simple testing strategy of the null hypothesis. To describe this result, consider first another assumption that is plausible in a blinded trial:

Assumption 1.5. (No treatment effect on exposure in the unexposed).

\begin{align*} & E_{1}^{a=0}=E_{1}^{a=1}&&\textrm{and} && E_{2}^{a=0, e_{1}=0}=E_{2}^{a=1, e_{1}=0} \end{align*}

Assumption 1.5 requires that there is no effect of treatment on time 1 exposure and on time 2 exposure following isolation. Conceptually, there is no causal path from *A* to $E_{1}$. Furthermore, there is no causal path from *A* to $E_{2}$ except through $E_{1}$ or $\Delta Y_{1}$. In a blinded trial, the exposure-seeking behavior at time 1 should not be a function of the actual treatment received. Yet, at time 2, those who developed the outcome at time 1, might reduce their contacts, for example, to minimize the spread of the disease. If the vaccine has an effect on the outcome at time 1, then the time 2 exposure might decrease at an increased rate among the placebo recipients. However, under isolation, the treatment would not affect their survival, by Assumption 1.1. Therefore, if the treatment is blinded, the time 2 exposure would be the same under both treatment and no treatment, making Assumption 1.5 plausible.

Assumption 1.5 is less restrictive than common assumptions in infectious disease epidemiology. In particular, random mixing is often assumed, which would require unconditional independence between the treatment and the exposures at both times, which implies Assumption 1.5. Moreover, under random mixing, the two exposures cannot be confounded; this is a restriction that Assumption 1.5 does not impose. Consider also the following exchangeability condition:

Assumption 1.6. (Exposure exchangeability).
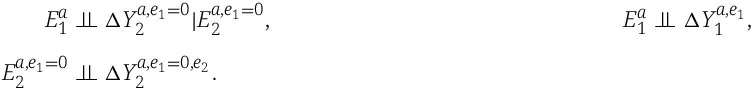

Exchangeabilities between the potential outcomes and the time 1 exposure are guaranteed by the lack of exposure-outcome confounders. However, in practice, exposure at time 1 and time 2 can be confounded by, for example, behavioral patterns. Thus, to make the independence between $E_{1}^{a}$ and $\Delta Y_{2}^{a, e_{1}=0}$ plausible, we have to control for the time 2 exposure. Moreover, 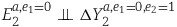 is equivalent to 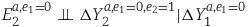 under Assumption 1.1. Conceptually, this independence holds in the absence of unmeasured exposure-outcome confounding. For ease of exposition, we present our results and assumptions without conditioning on measured covariates (confounders). However, the results generalize straightforwardly to settings with baseline covariates, which make the identifying assumptions more plausible in many applications (see Appendix S3 for details). Even when the measured baseline variables are conditioned on, some confounders that affect both the exposures and the outcomes can remain unmeasured. The more general Assumption 7 can be violated when we fail to adjust for such confounders. However, the resulting bias is expected to be modest when the influence or prevalence of the unmeasured confounder is limited. When the investigators suspect substantial bias due to unmeasured confounding, they can mitigate such effects using negative control outcomes, such as nonvaccine-targeted infections (see [1]). Adjustment for latent confounding is also motivated by the correlated-infections framework of Farrington et al. [4] and Unkel et al. [23], which show that the correlations between infections within individuals can be informative about the extent of heterogeneity in effective contact rates, that is, heterogeneity in exposure.

Under the directed acyclic graph (DAG) proposed by Janvin and Stensrud [12], Assumption 1.6 holds. See Appendix S5 for a replication of the DAG and a discussion on the Assumptions in [12].

The four assumptions introduced so far allow us to derive a simple equality that motivates our hypothesis test:

Proposition 1.7. (Invariant incidence rates).Suppose that Assumptions 1.1-1.6 hold. Under Hypothesis 1.3  

\begin{align*} & IR_1:= \frac{E[\Delta Y_1|A=1]}{E[\Delta Y_1|A=0]} = \frac{E[\Delta Y_2|A=1]}{E[\Delta Y_2|A=0]}:=IR_2. \end{align*}

See Proof in Appendix S6.

Thus, Proposition 1.7 allows us to test Hypothesis 1.3 by comparing incidence ratios ($IR_{1}$, $IR_{2}$). Under Assumptions 1.1-1.6, the test is only informative about the existence of waning, but not the extent of it. However, if a further monotonicity assumption is imposed on the potential outcomes over time, a direct correspondence can be established between the test statistics and the ratio $\left (1-VE_{2}^{\textrm{challenge}}\right )/\left (1-VE_{1}^{\textrm{challenge}}\right )$, which provides a measure of the extent of waning. See Appendix S7 for details.

We emphasize that incidence ratios are not HRs, because time-varying HRs are often used in the literature when studying the waning of a vaccine. In a discrete-time setting, the observed HRs are 


\begin{align*} &HR_{1}:= \frac{E[\Delta Y_{1}|A=1]}{E[\Delta Y_{1}|A=0]} && HR_{2}:=\frac{E[\Delta Y_{2}|\Delta Y_{1}=0, A=1]}{E[\Delta Y_{2}|\Delta Y_{1}=0, A=0]}. \end{align*}

At the first time, all individuals are event-free, regardless of which treatment arms they were randomized to. Thus, $IR_{1}=HR_{1}$. By Proposition 1.7, under the null hypothesis $HR_{1}=HR_{2}$, if and only if $P(\Delta Y_{1}=0|A=0)=P(\Delta Y_{1}=0|A=1)$, that is, the vaccine has no population-level effect on survival at the first time. Assuming that no effect of the vaccine is observed at the beginning of the study is unrealistic. Therefore, a change in the *HR* over time, unlike a change in the *IR* over time, is not a measure of waning. We quantify the bias of studying *HRs* instead of *IRs* in Appendix S8 and illustrate the performance of the HR-based testing in Appendix S9.

The test itself is suitable for assessing the presence of waning, however, it is generally uninformative of the magnitude of waning. In Appendix S2, we discuss the interpretation of the ratio of the incidence ratios when the alternative of the null hypothesis holds, and possible improvements on the bounds of Janvin and Stensrud [12] when waning is present.

## Estimation and data analysis

The test can be implemented, using summary data only (see Appendix S10 for details). Thus, most published vaccine trials can be reanalyzed, given that they have the incidence recorded at least at two times. First, we revisit the BNT162b2 trial analyzed by Janvin and Stensrud [12] and show that our test is able to detect waning in the same setting in which they could not claim waning previously. Then we give an example where the test does not reject the null hypothesis, and argue that the test result agrees with biological explanations, illustrated by a *Helicobacter pylori* vaccine study.

The authors in Janvin and Stensrud [12] have analyzed a blinded RCT, published by Thomas et al. [22], in which trial participants were randomized to receive a placebo (*A=0*) or the BNT162b2 COVID-19 vaccine (*A=1*). We are following the same division of the follow-up to the first and second interval as Janvin and Stensrud [12] in their main analysis, and an identical estimation procedure of the conditional expectation $E[Y_{k}|A=a]$ for $a \in \{0,1\}, \, k \in \{1,2\}$, where $Y_{k}$ is the cumulative incidence by the end of interval *k*. Since their assumptions only allowed partial identification of $VE_{2}^{\textrm{challenge}}$, they compared the point-identified $\widehat{VE}_{1}^{\textrm{challenge}}$ to the estimated lower and upper bounds of the second interval challenge effects, $\widehat{\mathcal{L}}, \widehat{\mathcal{U}}$, respectively. Although their point estimates suggested a waning effect of the vaccine, the null hypothesis of no waning could not have been rejected.

In comparison, the point estimate of $\widehat{IR}_{1}/\widehat{IR}_{2}$ is *0.48*  $\big (P=0.01,\; 95\%\ \textrm{CI}\ [0.279, 0.825]\big )$, thus clearly rejecting our null hypothesis of no change in the potential outcome of an individual upon exposure over time, at the *5%* level. Unless the condition in Remark 2 holds, that is, that the number of people for whom the vaccine’s effect improves over time equals the number for whom its efficacy decreases, the rejection can be interpreted as evidence of a change in the population challenge effect over time, that is, waning VE.

Consider now a second example. Zeng et al. [24] performed a double-blind, placebo-controlled RCT, to assess the efficacy, safety and immunogenicity of the Helicobacter pylori vaccine among children in China. The trial participants were randomized 1:1 to the two arms and 99% of them completed the 3-dose vaccination schedule. The primary analysis was performed among these participants (per-protocol-analysis) to assess the H pylori infection in the first 12 months, following the completion of the vaccine schedule.

Incidence data has been published at months 4,8,12,24, and 36. To compare with the primary analysis, we compared the incidence ratio until month 8 ($IR_{1}$), to the incidence ratio between month 8 and month 12 ($IR_{2}$). The choice of time intervals is application-specific and should be guided by the scientific question and, where relevant, public-health considerations. For example, decision-makers may wish to assess waning over periods aligned with feasible booster schedules. If booster doses can be administered every 6 months, one relevant comparison is VE during 0-6 months versus 6-12 months [14]. In the first period, there were 36 and 10 events for 1416 and 1403.6 person-years at risk in the placebo and treatment arms, respectively. Following that, in the second period, there were 14 and 4 events for 673.6 and 670.7 person-years at risk in the placebo and treatment arms, respectively. Using the direct *δ *-method, we derive the estimate for $\widehat{IR}_{1}/\widehat{IR}_{2} = 0.977$ (*P=0.97*  *95%CI*  *[0.263, 3.63]*).

The test statistic agrees with the findings of Zeng et al. [24]; they have found that VE is $72\%\;(95\%CI$  $[42.4\%, 87.6\%])$ at month 8 and $71.8\%\;(95\%CI$  $[48.2\%, 85.6\%])$ at month 12. Even though we argued that the naive VE should not be used for assessing vaccine waning due to the selection-bias introduced, their analysis of the incidence also points towards no waning.

In practice, arguments about waning are often based on the decay of immunological responses, measured by, for example, antibody levels. Zeng et al. [24] measured the serum-specific antibodies in the vaccine and the placebo group. While at baseline, the concentrations were similar in the 2 treatment groups and the vaccination elicited a strong immune response. The concentration of the serum-specific antibodies did not decrease significantly over time, agreeing with our test results.

## Discussion

The challenge effect quantifies changes in the protective effect of the vaccine. However, it is not possible to point identify the challenge effect from conventional RCTs, and the sharp bounds of partial identification can often be too wide to make claims about waning.

We show that instead of identifying the challenge effect, a sharp null hypothesis of no waning can be assessed accurately, with minimal assumptions; that is, unless some unrealistic cancellations of waning hold, we can interpret the null hypothesis as equivalent to the population-level notion of no waning, $VE_{1}^{\textrm{challenge}}\neq VE_{2}^{\textrm{challenge}}$. We derive a simple test statistic for the null hypothesis, which can be computed using the observed data from a standard RCT. In the absence of patient-level data, the test statistics can be computed using summary data only.

The test is designed to assess the existence of waning. When waning is confirmed by the test, further steps can be taken to try to determine the magnitude of waning. This could, for example, be a follow-up trial, a reanalysis of the existing data with methods building on the results from Janvin and Stensrud [12], or additional analyses leveraging expert knowledge on exposure probabilities, as discussed in Appendix S2.1.

When evidence of waning remains inconclusive, this may reflect the limited duration of follow-up. Such limitations are common in vaccine trials, where the blinded phase is often discontinued once early efficacy has been established. A next step is to extend the proposed testing strategy to incorporate information from, for example, post-unblinding and open-label follow-up period [5], or to adapt the strategy to modified trial designs, for example, inspired by Follmann et al. [6]. These extensions can allow us to relax some assumptions and improve power by leveraging additional cases.

## Supplementary Material

Web_Material_kwag118

## Data Availability

The real data underlying this article are available as summary data in the referenced manuscripts. The simulated data are reproducible using the code provided in the article’s online supplementary material.
