# Supplementary material for: A simple and powerful test of vaccine waning
Source: Am J Epidemiol. 2026 Jul 1;195(9):2469–75. doi: 10.1093/aje/kwag118 (PMC13537855; doi:10.1093/aje/kwag118)
Supplement: Supplementary file 1 [file web_material_kwag118.zip › A simple and powerful test of vaccine waning_appendix.pdf]

Supplementary Materials for A simple and powerful test of vaccine waning

Gellért Perényi, Matias Janvin, Mats J. Stensrud

Included Supplementary Materials

List of Appendices

- Appendix S1: Sensitivity analysis of the depletion of susceptibles
- Appendix S2: Interpretation of the IR, under the alternative
  - Appendix S2.1: Sensitivity analysis on the challenge effects
- Appendix S3: Conditional waning
- Appendix S4: Principal Strata at Different Times
- Appendix S5: On the Assumptions of Janvin and Stensrud (2024),
- Appendix S6: Proof of Proposition 1
- Appendix S7: Interpretation of the p-value under parametric assumptions
- Appendix S8: Hazard-ratio based waning under the null hypothesis
- Appendix S9: Simulation
  - Appendix S9.1: Sensitivity analysis
  - Appendix S9.2: Determining transition rates
- Appendix S10: Variance estimation

List of Figures

- Figure S1
- Figure S2
- Figure S3
- Figure S4
- Figure S5
- Figure S6
- Figure S7
- Figure S8

S1 Sensitivity analysis of the depletion of susceptibles

In this section, we present a sensitivity analysis that uses investigators' beliefs about the extent of depletion of susceptible individuals to examine the plausibility of the null hypothesis of no waning. Following the main text, we present our results in a setting with 2 intervals; however, extension to a setting with K intervals follows straightforwardly. As in Janvin and Stensrud [1], we define the parameter ψ as the ratio of the expected potential outcomes under treatment at the two timepoints, in a challenge trial:

$$\psi := \frac{E[\Delta Y_1^{a=1,e_1=1}]}{E[\Delta Y_2^{a=1,e_1=0,e_2=1}]}.$$

Suppose that no waning of the placebo holds, at least in the population sense, that is

$$E[\Delta Y_1^{a=0,e_1=1}] = E[\Delta Y_2^{a=0,e_1=0,e_2=1}], \tag{1}$$

then

$$\psi = \frac{1 - VE_1^{\text{challenge}}}{1 - VE_2^{\text{challenge}}}.$$

Observing $\psi \neq 1$ shows that the challenge effect changed over time, thus the vaccine has waned in either direction. Janvin and Stensrud [1] describe the challenge trial corresponding to the estimand ψ, which bears similarities to the trials using delayed vaccination to assess waning, when the infectious agent enters the population only after a certain calendar time [2, 3].

The following proposition decomposes the challenge effect into a ratio of hazard ratios (HR ratio) and an error term that reflects the departure of the challenge effect from the HR ratio.

Proposition 2. *Under Assumptions 1, 3, 4, 12, 13, 14, and* (1)

$$\psi = \frac{E[\Delta Y_1 \mid A = 1]}{E[\Delta Y_1 \mid A = 0]} \times \frac{E[\Delta Y_2 \mid \Delta Y_1 = 0, A = 0]}{E[\Delta Y_2 \mid \Delta Y_1 = 0, A = 1]} \\ \times \frac{E[\Delta Y_2^{e_1=0} \mid A = 0]/E[\Delta Y_2 \mid \Delta Y_1 = 0, A = 0]}{E[\Delta Y_2^{e_1=0} \mid A = 1]/E[\Delta Y_2 \mid \Delta Y_1 = 0, A = 1]}. \tag{2}$$

The proof of Proposition 2 follows from the proof of Theorem 1 in Janvin and Stensrud [1]. The error term in the second line of (2) can differ from 1 due to (*i*) differential depletion of susceptible individuals over time and (*ii*) differential acquisition of natural immunity from undetected infections in interval 1 across treatment arms. In the absence of (*i*) and (*ii*), the challenge effect is equal to the HR ratio, as formalized in Proposition 1 of Janvin and Stensrud [1]. When data is available on infectious outcomes not targeted by the treatment, that is, negative control outcomes, the bias introduced by depletion of susceptibles can be assessed using methods introduced by Ashby et al. [4]. Consider the two-interval setting introduced in the main text, where in addition to the primary outcome, ΔN_k indicates whether the negative control outcome occurs during interval k. Then, adapting the estimand introduced by Ashby et al. [4], the relative change in susceptibility between the two arms can be measured by

$$\frac{E[\Delta N_2 | \Delta Y_1 = \Delta N_1 = 0, A = 1]}{E[\Delta N_2 | \Delta Y_1 = \Delta N_1 = 0, A = 0]}.$$

Under suitable assumptions on the confounding structure, this quantity can be used to debias the hazard ratio, thereby improving our ability to detect waning. Even though for similar outcomes such as COVID-19 infection and other acute respiratory infections, it may be reasonable to assume that the same unmeasured variables act as confounders for both outcomes, the functional form of their effects may differ across outcomes. Thus, while exact results on identification require strong and untestable assumptions, the approach of Ashby

et al. [4] may still be useful as an exploratory tool for detecting residual confounding.

Next, we will describe a sensitivity analysis that uses an investigator's belief about the size of the error term in (2). Suppose an investigator believes that

$$1 \leq \frac{E[\Delta Y_2^{e_1=0} \mid A=a]}{E[\Delta Y_2 \mid \Delta Y_1 = 0, A=a]} \leq \alpha \tag{3}$$

for all $a \in \{0,1\}$. In words, the investigator believes that isolation during interval 1, which retains susceptible individuals in the risk set and prevents natural immunity from undetected infections, can at most lead to an α-fold increase in infections during interval 2 in either treatment group. The lower bound in (3) is a modest tightening of the bound in Assumption 4 (exposure effect restriction) of Janvin and Stensrud [1] reflecting that isolation does not make the population at risk more protected against infectious exposures in the future.

Proposition 3 (Sensitivity analysis)**.** *Suppose Assumptions 1, 3, 4, 12, 13, 14 and* (1), (3) *hold. Next, suppose that* $\psi = 1$*, i.e. the null hypothesis of no waning holds in expectation. Then*

$$\left(\frac{E[\Delta Y_1 \mid A=1]}{E[\Delta Y_1 \mid A=0]} \times \frac{E[\Delta Y_2 \mid \Delta Y_1 = 0, A=0]}{E[\Delta Y_2 \mid \Delta Y_1 = 0, A=1]} \right)^{-1} \leq \alpha \ . \tag{4}$$

Expression (4) follows directly from using (3) and $\psi = 1$ in (2). The left hand side of (4) is the (reciprocal) HR ratio, which is only a functional of the observed data, whereas α is the investigator's belief about the maximal possible value of the ratios $E[\Delta Y_2^{e_1=0} \mid A = a]/E[\Delta Y_2 \mid \Delta Y_1 = 0, A = a]$ for $a \in \{0,1\}$. If the investigator's chosen value of α is too small to satisfy (4), we obtain a contradiction: the observed HR ratio cannot alone be explained by the investigators' belief (α) about the depletion of susceptible individuals.

For example, suppose that

$$\alpha = 5,$$

$$E[\Delta Y_1 \mid A = 1] = 0.01, \quad E[\Delta Y_1 \mid A = 0] = 0.1,$$

$$E[\Delta Y_2 \mid \Delta Y_1 = 0, A = 1] = E[\Delta Y_2 \mid \Delta Y_1 = 0, A = 0] = 0.1,$$

then Proposition 3 gives the contradiction $10 \leq 5$. Whenever (4) leads to a contradiction, this implies that the premises assumed in Proposition 3 are false: in particular, the null hypothesis of no waning ($\psi = 1$) may be implausible in light of the investigators' belief (α). Although it is not obvious how investigators can agree on a particular choice of α, Proposition 3 offers a way to quantitatively state beliefs about the depletion of susceptible individuals and use these to assess the plausibility of the null hypothesis of no waning.

S2 Interpretation of the IR, under the alternative

Suppose that Hypothesis 0 does not hold, that is, $\Delta Y_2^{a,e_1=0,e_2=1}$ and $\Delta Y_1^{a,e_1=1}$ can potentially differ. Under Assumptions 1 and 3, by identical arguments as in Appendix S6, we can show that $IR_1 = 1 - VE_1^{\text{challenge}}$. Since $IR_1 = HR_1$, our result agrees with the point identification claim of Janvin and Stensrud [1].

By the same reasoning as in Appendix S6, we can show that under Assumptions 1-4, 12-14

$$\begin{aligned} E[\Delta Y_2 | A = a] = {} & E[\Delta Y_2^a | E_1^a = 0] P(E_1^a = 0) \\ & + E[\Delta Y_2^a | E_1^a = 1] P(E_1^a = 1), \end{aligned}$$

and that

$$E[\Delta Y_2^a | E_1^a = 0] P(E_1^a = 0) = E[\Delta Y_2^{a,e_1=0,e_2=1}] P(E_2^{a,e_1=0} = 1, E_1^a = 0).$$

However, under the alternative of the null hypothesis $E[\Delta Y_2^a | E_1^a = 1] P(E_1^a = 1)$ is not necessarily equal to 0. In addition to the previously presented assumptions, suppose the following:

Assumption 5 (Extended exposure exchangeability)**.**

$$E_1^a \perp\!\!\!\perp \Delta Y_2^{a,e_1=1} | \Delta Y_1^{a,e_1=1} = 0, E_2^{a,e_1=1}$$

$$\text{and} \qquad E_2^{a,e_1=1} \perp\!\!\!\perp \Delta Y_2^{a,e_1=1,e_2} | \Delta Y_1^{a,e_1=1} = 0$$

Assumption 5 generalizes Assumption 4 such that it holds under the intervention $e_1 = 1$. While previously, by Assumption 1, the intervention $e_1 = 0$ resulted in implicit conditioning on $\Delta Y_1^{a,e_1=0} = 0$, since under $e_1 = 1$, the time 1 outcome is not deterministically 0, we have to condition on it explicitly. Both Assumptions 4 and 5 hold under the DAG proposed by Janvin and Stensrud [1], reproduced in Figure S2.

Continuing with Equation (12)

$$E[\Delta Y_2^{a,e_1=1}|E_1^a=1,\Delta Y_1^{a,e_1=1}=0,E_2^{a,e_1=1}=1]$$

$$\times P(E_2^{a,e_1=1}=1|E_1^a=1,\Delta Y_1^{a,e_1=1}=0)P(\Delta Y_1^{a,e_1=1}=0|E_1^a=1)P(E_1^a=1)$$

$$\overset{\text{Assumption 5}}{=} E[\Delta Y_2^{a,e_1=1}|\Delta Y_1^{a,e_1=1}=0,E_2^{a,e_1=1}=1]$$

$$\times P(E_2^{a,e_1=1}=1|E_1^a=1,\Delta Y_1^{a,e_1=1}=0)P(\Delta Y_1^{a,e_1=1}=0|E_1^a=1)P(E_1^a=1)$$

$$\overset{\text{Assumption 12}}{=} E[\Delta Y_2^{a,e_1=1,e_2=1}|\Delta Y_1^{a,e_1=1}=0,E_2^{a,e_1=1}=1]$$

$$\times P(E_2^{a,e_1=1}=1|E_1^a=1,\Delta Y_1^{a,e_1=1}=0)P(\Delta Y_1^{a,e_1=1}=0|E_1^a=1)P(E_1^a=1)$$

$$\overset{\text{Assumption 5}}{=} E[\Delta Y_2^{a,e_1=1,e_2=1}|\Delta Y_1^{a,e_1=1}=0]P(E_2^{a,e_1=1}=1|E_1^a=1,\Delta Y_1^{a,e_1=1}=0)$$

$$\times P(\Delta Y_1^{a,e_1=1}=0|E_1^a=1)P(E_1^a=1)$$

$$\overset{\text{Assumption 4}}{=} E[\Delta Y_2^{a,e_1=1,e_2=1}|\Delta Y_1^{a,e_1=1}=0]P(\Delta Y_1^{a,e_1=1}=0)$$

$$\times P(E_2^{a,e_1=1}=1|E_1^a=1,\Delta Y_1^{a,e_1=1}=0)P(E_1^a=1)$$

$$\overset{\text{Assumption 12}}{=} E[\Delta Y_2^{a,e_1=1,\delta y_1=0,e_2=1}|\Delta Y_1^{a,e_1=1}=0]P(\Delta Y_1^{a,e_1=1}=0)$$

$$\times P(E_2^{a,e_1=1}=1|E_1^a=1,\Delta Y_1^{a,e_1=1}=0)P(E_1^a=1)$$

$$\overset{\text{Assumption 2}}{=} E[\Delta Y_2^{a,e_1=0,e_2=1}|\Delta Y_1^{a,e_1=1}=0]P(\Delta Y_1^{a,e_1=1}=0)$$

$$\times P(E_2^{a,e_1=1}=1|E_1^a=1,\Delta Y_1^{a,e_1=1}=0)P(E_1^a=1)$$

$$= P(\Delta Y_2^{a,e_1=0,e_2=1}=1,\Delta Y_1^{a,e_1=1}=0)$$

$$\times P(E_2^{a,e_1=1}=1|E_1^a=1,\Delta Y_1^{a,e_1=1}=0)P(E_1^a=1)$$

Under the null hypothesis, the joint probability,

$$P(\Delta Y_2^{a,e_1=0,e_2=1}=1,\Delta Y_1^{a,e_1=1}=0)$$

is equal to 0; however, under the alternative, not necessarily.

Therefore,

$$E[\Delta Y_2|A=a] = P(\Delta Y_2^{a,e_1=0,e_2=1}=1)P(E_2^{a,e_1=0}=1|E_1^a=0,\Delta Y_1^{a,e_1=0}=0)P(E_1^a=0)$$
$$+ P(\Delta Y_2^{a,e_1=0,e_2=1}=1,\Delta Y_1^{a,e_1=1}=0)P(E_2^{a,e_1=1}=1|E_1^a=1,\Delta Y_1^{a,e_1=1}=0)P(E_1^a=1) \tag{5}$$

and

$$IR_2 = \frac{P(\Delta Y_2^{a=1,e_1=0,e_2=1}=1)p_0^{2,a=1}p_0^1 + P(\Delta Y_2^{a=1,e_1=0,e_2=1}=1,\Delta Y_1^{a=1,e_1=1}=0)p_1^{2,a=1}p_1^1}{P(\Delta Y_2^{a=0,e_1=0,e_2=1}=1)p_0^{2,a=0}p_0^1 + P(\Delta Y_2^{a=0,e_1=0,e_2=1}=1,\Delta Y_1^{a=0,e_1=1}=0)p_1^{2,a=0}p_1^1},$$

where $p_{e_1}^{2,a} = P(E_2^{a,e_1}=1|E_1^a=e_1,\Delta Y_1^{a,e_1}=0)$ and $p_{e_1}^1 = P(E_1^a=e_1) \overset{\text{Assumption 3}}{=} P(E_1=e_1)$.

Depending on the exposure probabilities, $P(\Delta Y_2^{a,e_1=0,e_2=1}=1)$, and $P(\Delta Y_2^{a,e_1=0,e_2=1}=1,\Delta Y_1^{a,e_1=1}=0)$, IR_2 can be either below or above the $VE_2^{\text{challenge}}$, thus IR_2 is uninformative about the extent of the waning.

Assumption 6 (Sharp no waning of the placebo)**.**

$$\Delta Y_2^{a=0,e_1=0,e_2=1} = \Delta Y_1^{a,e_1=1} \quad \text{w.p } 1.$$

Assumption 6 was used to establish equivalence between individual and population waning in Remark 2. Under the alternative and Assumption 6, we allow for $\Delta Y_2^{a=1,e_1=0,e_2=1} \neq \Delta Y_1^{a=1,e_1=1}$ with a non-zero probability.

By Assumption 6

$$\begin{aligned}
IR_2 &= \frac{P(\Delta Y_2^{a=1,e_1=0,e_2=1}=1)p_0^{2,a=1}p_0^1 + P(\Delta Y_2^{a=1,e_1=0,e_2=1}=1,\Delta Y_1^{a=1,e_1=1}=0)p_1^{2,a=1}p_1^1}{P(\Delta Y_2^{a=0,e_1=0,e_2=1}=1)p_0^{2,a=0}p_0^1} \\
&= \frac{P(\Delta Y_2^{a=1,e_1=0,e_2=1}=1)p_0^{2,a=1}p_0^1}{P(\Delta Y_2^{a=0,e_1=0,e_2=1}=1)p_0^{2,a=0}p_0^1} + \frac{P(\Delta Y_2^{a=1,e_1=0,e_2=1}=1,\Delta Y_1^{a=1,e_1=1}=0)p_1^{2,a=1}p_1^1}{P(\Delta Y_2^{a=0,e_1=0,e_2=1}=1)p_0^{2,a=0}p_0^1} \\
&= 1 - VE_2^{\text{challenge}} + \frac{P(\Delta Y_2^{a=1,e_1=0,e_2=1}=1,\Delta Y_1^{a=1,e_1=1}=0)p_1^{2,a=1}p_1^1}{P(\Delta Y_2^{a=0,e_1=0,e_2=1}=1)p_0^{2,a=0}p_0^1}.
\end{aligned}$$

In other words, under Assumption 6 and the alternative, the second timepoint incidence ratio overestimates $1 - VE_2^{\text{challenge}}$. The size of the overestimation depends on the exposure probabilities and the size of the principal strata, which corresponds to individuals who transitioned from immune to harmed or from helped to doomed. Those who experienced the improvement of the vaccine over time (doomed to helped and harmed to immune) do not add to the bias, since even if they remained event-free in time 1, in the second interval they do not develop the outcome under treatment, that is the same outcome that they would have experienced, had they been isolated at time 1.

Due to the sign of the bias, when $IR_1 < IR_2$, that is, based on the incidence ratios, the vaccine seemingly wanes, $VE_1^{\text{challenge}} < VE_2^{\text{challenge}}$ can still hold. The qualitative difference between the incidence ratio and the challenge effects, under Assumption 6, can occur if

$$P(\Delta Y_2^{a=1,e_1=0,e_2=1} = 0, \Delta Y_1^{a=1,e_1} = 1) > P(\Delta Y_2^{a=1,e_1=0,e_2=1} = 1, \Delta Y_1^{a=1,e_1} = 0),$$

that is, the probability of the effect of the individual vaccine improvement is greater than the probability of individual waning, and

$$\begin{aligned} &P(\Delta Y_2^{a=1,e_1=0,e_2=1} = 1, \Delta Y_1^{a=1,e_1} = 0)(p_0^{2,a=1}p_0^1 + p_1^{2,a=1}p_1^1) \\ &> P(\Delta Y_2^{a=1,e_1=0,e_2=1} = 0, \Delta Y_1^{a=1,e_1} = 1)p_0^{2,a=1}p_0^1. \end{aligned}$$

Assuming independence between the exposures, given survival ($E_1^a \perp\!\!\!\perp E_2^{a,e_1} | \Delta Y_1^{a,e_1} = 0$, similar to Equation 7 of Janvin and Stensrud [1]), $p_1^{2,a} = p_0^{2,a}$ follows and the second condition simplifies to

$$P(\Delta Y_2^{a=1,e_1=0,e_2=1} = 1, \Delta Y_1^{a=1,e_1} = 0) > P(\Delta Y_2^{a=1,e_1=0,e_2=1} = 0, \Delta Y_1^{a=1,e_1} = 1)p_0^1.$$

Therefore, if the extent of waning is substantial compared to vaccine improvement and the

infectious pressure at time 1 is high, waning could be mistakenly claimed based on the incidence ratios, even though the challenge effect has increased over time.

If $IR_2 < IR_1$, then due to the positive bias of IR_2, $VE_2^{\text{challenge}} > VE_1^{\text{challenge}}$, that is, if the vaccine seemingly improved based on the incidence ratios, indeed the vaccine improved in the challenge effect sense. However, the size of this improvement is overestimated by the incidence ratios, with the magnitude of the overestimation depending on the probability of vaccine waning and the exposure probabilities.

S2.1 Sensitivity analysis on the challenge effects

From Equation (5) it follows that

$$P(\Delta Y_2^{a,e_1=0,e_2=1} = 1)p_0^{2,a}p_0^1 = E[\Delta Y_2|A = a] - P(\Delta Y_2^{a,e_1=0,e_2=1} = 1, \Delta Y_1^{a,e_1=1} = 0)p_1^{2,a}p_1^1.$$

Under Assumption 3 and 12, $p_0^{2,a=1}p_0^1 = p_0^{2,a=0}p_0^1$, thus

$$1 - VE_2^{challenge} = \frac{E[\Delta Y_2|A = 1] - P(\Delta Y_2^{a=1,e_1=0,e_2=1} = 1, \Delta Y_1^{a=1,e_1=1} = 0)p_1^{2,a=1}p_1^1}{E[\Delta Y_2|A = 0] - P(\Delta Y_2^{a=0,e_1=0,e_2=1} = 1, \Delta Y_1^{a=0,e_1=1} = 0)p_1^{2,a=0}p_1^1}. \quad (6)$$

Using information from the observed data and Assumption 6, we can bound some of the expressions in Equation (6). As

$$\begin{aligned} E[Y_2|A = a] = &\big(P(\Delta Y_2^{a,e_1=0,e_2=1} = 1, \Delta Y_1^{a,e_1=1} = 0) + P(\Delta Y_2^{a,e_1=0,e_2=1} = 1, \Delta Y_1^{a,e_1=1} = 1)\big)p_0^2p_0^1 \\ &+ P(\Delta Y_2^{a,e_1=0,e_2=1} = 1, \Delta Y_1^{a,e_1=1} = 0)p_1^2p_1^1, \end{aligned}$$

thus

$$P(\Delta Y_2^{a,e_1=0,e_2=1} = 1, \Delta Y_1^{a,e_1=1} = 0) \leq \frac{E[\Delta Y_2|A = a]}{p_0^2p_0^1 + p_1^2p_1^1}. \quad (7)$$

Under Assumption 6 $P(\Delta Y_2^{a=0,e_1=0,e_2=1}=1, \Delta Y_1^{a=0,e_1=1}=0)=0$, thus

$$E[\Delta Y_1|A=0] = P(\Delta Y_2^{a=0,e_1=0,e_2=1}=1, \Delta Y_1^{a=0,e_1=1}=1)p_1^1$$

and

$$E[\Delta Y_2|A=0] = P(\Delta Y_2^{a=0,e_1=0,e_2=1}=1, \Delta Y_1^{a=0,e_1=1}=1)p_0^2(1-p_1^1),$$

implying that the equality

$$\frac{E[\Delta Y_1|A=0]}{E[\Delta Y_2|A=0]} = \frac{p_1^1}{p_0^2(1-p_1^1)} \tag{8}$$

must hold.

Since under Assumption 6

$$VE_2^{\text{challenge}} = 1 - IR_2 + \frac{P(\Delta Y_2^{a=1,e_1=0,e_2=1}=1, \Delta Y_1^{a=1,e_1=1}=0)p_1^2p_1^1}{E[\Delta Y_2|A=0]},$$

$VE_2^{\text{challenge}}$ is maximized when $P(\Delta Y_2^{a=1,e_1=0,e_2=1}=1, \Delta Y_1^{a=1,e_1=1}=0)p_1^2p_1^1 - E[\Delta Y_2|A=1]$ reaches its maximum. By Equation (7), the maximum is reached at $P(\Delta Y_2^{a=1,e_1=0,e_2=1}=1, \Delta Y_1^{a=1,e_1=1}=0) = \frac{E[\Delta Y_2|A=1]}{p_0^2p_0^1+p_1^2p_1^1}$, thus it is equal to

$$E[\Delta Y_2|A=1]\frac{p_1^2p_1^1 - p_0^2p_0^1 - p_1^2p_1^1}{p_0^2p_0^1+p_1^2p_1^1} = E[\Delta Y_2|A=1]\frac{-p_0^2p_0^1}{p_0^2p_0^1+p_1^2p_1^1}.$$

By Equation (8),

$$p_1^1 = \frac{E[\Delta Y_1|A=0]p_0^2}{E[\Delta Y_2|A=0]+E[\Delta Y_1|A=0]p_0^2} \quad \text{and} \quad p_0^1 = \frac{E[\Delta Y_2|A=0]}{E[\Delta Y_2|A=0]+E[\Delta Y_1|A=0]p_0^2},$$

thus, the maximum simplifies to

$$E[\Delta Y_2|A=1]\frac{-p_0^2E[\Delta Y_2|A=0]}{p_0^2E[\Delta Y_2|A=0]+p_1^2E[\Delta Y_1|A=0]p_0^2} = E[\Delta Y_2|A=1]\frac{-E[\Delta Y_2|A=0]}{E[\Delta Y_2|A=0]+p_1^2E[\Delta Y_1|A=0]}.$$

Since all expectations are non-negative, the fraction increases in p_1^2; therefore, it reaches

its maximum at $p_1^2 = 1$. Then,

$$
\begin{aligned}
VE_2^{\text{challenge}} &\leq 1 + \frac{E[\Delta Y_2|A=1]}{E[\Delta Y_2|A=0]} \frac{-E[\Delta Y_2|A=0]}{E[\Delta Y_2|A=0] + p_1^2 E[\Delta Y_1|A=0]}\Big|_{p_1^2=1} \\
&= 1 - \frac{E[Y_2|A=1]}{E[Y_1|A=0] + E[Y_2|A=0]},
\end{aligned}
$$

which is the same upper bound on the challenge effect as derived by Janvin and Stensrud [1]. However, if data are available from a standard RCT and there is knowledge about the probability $P(E_2^{a,e_1=1} = 1|E_1^a = 1, \Delta Y_1^{a,e_1=1} = 0)$, for example, from contact tracing studies, the upper bound can be considerably improved.

To illustrate the result, we took the observed quantities $E[Y_1|A=0], E[Y_1|A=1], E[Y_2|A=0]$, and $E[Y_2|A=1]$ from the BNT162b2 trial [5], and plotted the upper bound under the changing values of p_1^2 in Figure S1

S3 Conditional waning

The challenge effect, and thus waning, can be defined conditional on baseline covariates.

Definition 1 (Conditional challenge effect)**.** Denote the measured baseline covariates $\mathbf{L} \in \mathcal{L}$. Then the conditional challenge effects at times 1 and 2 are

$$
\begin{aligned}
VE_1^{\text{challenge}}(\mathbf{l}) &= 1 - \frac{E[\Delta Y_1^{a=1,e_1=1}|\mathbf{L}=\mathbf{l}]}{E[\Delta Y_1^{a=0,e_1=1}|\mathbf{L}=\mathbf{l}]}, \\
VE_2^{\text{challenge}}(\mathbf{l}) &= 1 - \frac{E[\Delta Y_1^{a=1,e_1=0,e_2=1}|\mathbf{L}=\mathbf{l}]}{E[\Delta Y_1^{a=0,e_1=0,e_2=1}|\mathbf{L}=\mathbf{l}]}
\end{aligned}
$$

Correspondingly, the incidence rates are

$$
IR_1(\mathbf{l}) := \frac{E[\Delta Y_1|A=1,\mathbf{L}=\mathbf{l}]}{E[\Delta Y_1|A=0,\mathbf{L}=\mathbf{l}]} \qquad IR_2(\mathbf{l}) := \frac{E[\Delta Y_2|A=1,\mathbf{L}=\mathbf{l}]}{E[\Delta Y_2|A=0,\mathbf{L}=\mathbf{l}]}
$$

Suppose that Assumption 3 holds only conditionally on the baseline covariates.

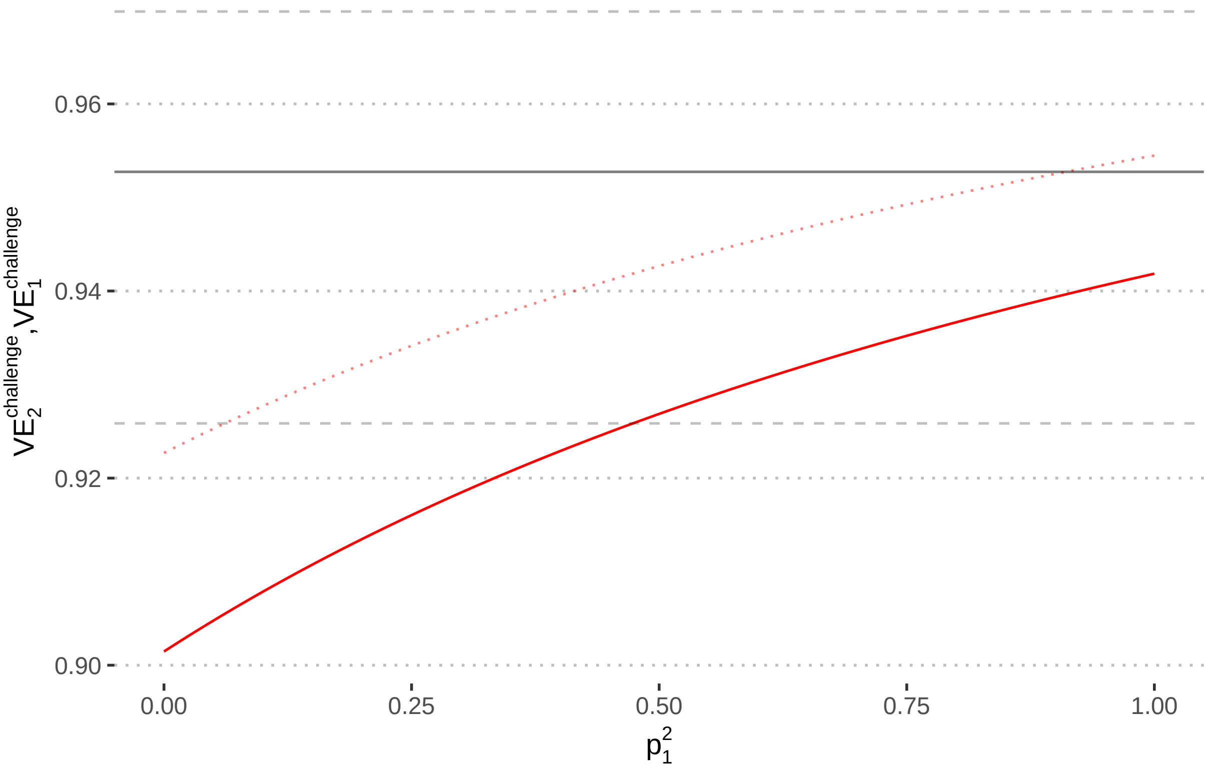

Figure S1: The changing value on the upper bound of $VE_2^{\text{challenge}}$ (solid red line), for some p_1^2, with the corresponding one-sides 95% confidence interval (dashed red). $VE_1^{\text{challenge}}$ (solid black), is point-identified, regardless of the value of p_1^2 or other exposure probabilities. A dashed black line represents the corresponding 95% two-sided confidence interval. The values at $p_1^2 = 1$ are equal to the findings of Janvin and Stensrud [1].

Assumption 7 (Conditional exposure exchangeability)**.**

$$E_1^a \perp\!\!\!\perp \Delta Y_2^{a,e_1=0} | E_2^{a,e_1=0}, \mathbf{L}, \qquad E_1^a \perp\!\!\!\perp \Delta Y_1^{a,e_1} | \mathbf{L},$$

$$E_2^{a,e_1=0} \perp\!\!\!\perp \Delta Y_2^{a,e_1=0,e_2} | \mathbf{L}.$$

For example, suppose that there are some *measured* confounders between the exposures and the outcome, such as age or physical fitness. Then, in order for the independence to hold between the outcomes and the exposures, all confounders should be conditioned on.

Similarly, if randomization occurs conditional on the baseline covariates, Assumption 13 and 14 hold conditionally.

Assumption 8 (Conditional treatment exchangeability)**.**

$$E_1^a, \Delta Y_1^a, \Delta Y_1^{a,e_1}, E_2^{a,e_1}, E_2^{a,e_1\delta y_1=0}, \Delta Y_2^{a,e_1,\delta y_1=0,e_2} \perp\!\!\!\perp A | \mathbf{L}$$

for all $a, e_1, e_2 \in \{0, 1\}$.

Assumption 9 (Conditional positivity)**.**

$$P(A = a | \mathbf{L} = \mathbf{l}) > 0$$

for all $a \in \{0, 1\}$, and for all $\mathbf{l} \in \mathcal{L}$, such that $P(\mathbf{L} = \mathbf{l}) > 0$.

Analogously to the proof in Appendix S6 we can show that under Hypothesis 0 and Assumptions 1-3 and 7

$$IR_1(\mathbf{l}) = IR_2(\mathbf{l}) \quad \forall \mathbf{l} \in \mathcal{L}$$

Then, the null hypothesis of no waning may be evaluated separately across subgroups; however, the analyses should control for multiple testing [6, 7].

Alternatively, assume constant proportionality across the subgroups defined by $\mathbf{L}$.

Assumption 10 (Constant incidence ratios)**.**

$$E[\Delta Y_1|A=1,\mathbf{L}=\mathbf{l}] = \alpha_1 E[\Delta Y_1|A=0,\mathbf{L}=\mathbf{l}]$$

$$E[\Delta Y_2|A=1,\mathbf{L}=\mathbf{l}] = \alpha_2 E[\Delta Y_2|A=0,\mathbf{L}=\mathbf{l}]$$

for all $\mathbf{l} \in \mathcal{L}$.

Under the null hypothesis and Assumptions 1 and 7, we have that $IR_1(\mathbf{l}) = IR_2(\mathbf{l})$. Thus, it follows that in this setting $\alpha_1 = \alpha_2$ must hold. Then

$$\frac{IR_1}{IR_2} = \frac{\int IR_1(\mathbf{l})\mathrm{d}P(\mathbf{L})}{\int IR_2(\mathbf{l})\mathrm{d}P(\mathbf{L})} = \int \frac{IR_1(\mathbf{l})}{IR_2(\mathbf{l})}\mathrm{d}P(\mathbf{L}) = \frac{\alpha_1}{\alpha_2}\int \frac{1}{1}\mathrm{d}P(\mathbf{L}) = 1,$$

therefore, the null hypothesis implies that there is no marginal population-level waning.

ctionTesting for multivalued exposure Assumption 12 relies on the binary categorization of the exposure. Certain individuals are unexposed, and all others are exposed according to some fixed intensity of exposure. Then the challenge effects, with respect to which waning is defined, are formulated in terms of this unspecified fixed exposure. However, in certain scenarios, defining exposure as binary, is implausible, for example, if multiple strains circulate in the population with different infectivity, the intensity of exposure varies according to the strains, or, when follow-up period spans over longer periods, certain individuals might face repeated exposures, which leads to a definition of a multi-valued exposure.

We revisit the findings of Janvin and Stensrud [1] in their Appendix D, which shows that under a stability assumption on the distribution of the "strength of exposure", conditional on being exposed, we can still test for the waning of the vaccine, using the partial-identification bounds derived. However, by not including in the null hypothesis the restriction of binary intensity, considerable power is lost.

Adopting the setup and the notation of Janvin and Stensrud [1], suppose that there exists a finer-scale version of the randomized trial described in the main text, denoted with G. In this modified challenge trial G, individuals are randomized to treatment or control ($A \in \{0, 1\}$) at baseline as before, and then at time 1 and time 2, to some intensity of exposure, denoted with $Q_1, Q_2 \in \mathcal{Q}$, respectively. $\mathcal{Q}$ can be interpreted as the conceivable range of the number of infectious particles per exposure, the number of exposures during the follow-up period, or the cross-product of the former two. Suppose that no exposure is well-defined for Q_1 and Q_2 as well, that is, there are no multiple versions of no exposure in the modified trial. In G, the coarse-grained exposure is defined as

$$E_k = \mathbb{I}(Q_k \neq 0) \text{ and } E_2^{q_1=0} = \mathbb{I}(Q_2^{q_1=0} \neq 0).$$

For brevity, we do not restate all the assumptions of Janvin and Stensrud [1], interested readers should see their Assumptions 9-12 in their Appendix D. However, we restate Proposition 2, which is central to our argument:

Proposition 4 (Restatement of Proposition 2 in Janvin and Stensrud [1])**.** *Under Assumptions 9-12 of Janvin and Stensrud [1] and Assumptions 12 (i) and 14 of this manuscript,*

$$E[\Delta Y_1^a | E_1^a = 1, A = a] = E_G[E_G[\Delta Y_1^{a,Q_1}] | E_1 = 1, A = a]$$

$$E[\Delta Y_2^{a,e_1=0} | E_2^{a,e_1=0} = 1, A = a] = E_G[E_G[\Delta Y_2^{a,q_1=0,Q_2^{q_1=0}}] | E_2^{q_1=0} = 1, A = a].$$

Under the further Assumptions 1, 3, 13 from our manuscript and Assumption 4 of Janvin and Stensrud [1],

$$1 - \frac{E_G[E_G[\Delta Y_1^{a=1,Q_1}]|E_1 = 1, A = 1]}{E_G[E_G[\Delta Y_1^{a=0,Q_1}]|E_1 = 1, A = 0]}] = 1 - \frac{E[\Delta Y_1|A = 1]}{E[\Delta Y_1|A = 0]} \tag{9}$$

$$1 - \frac{E_G[E_G[\Delta Y_1^{a=1,q_1=0,Q_2^{q_1=0}}]|E_2^{q_1=0} = 1, A = 1]}{E_G[E_G[\Delta Y_1^{a=0,q_1=0,Q_2^{q_1=0}}]|E_2^{q_1=0} = 1, A = 0]}] \in \left[1 - \frac{E[\Delta Y_1 + \Delta Y_2|A = 1]}{E[\Delta Y_2|A = 0]}, 1 - \frac{E[\Delta Y_2|A = 1]}{E[\Delta Y_1 + \Delta Y_2|A = 0]}\right] \tag{10}$$

See proof of the proposition in Appendix D of Janvin and Stensrud [1].

Janvin and Stensrud [1] provided an interpretation of equations (9)-(10): the right hand sides are identification formulas for the ratio of expected potential outcomes, under exposure intervention in which $e_k = 0$ corresponds to isolation, and $e_k = 1$ corresponds to an exposure with randomized intensity, where the investigator draws the intensity Q_1 and $Q_2^{q_1=0}$ from the distribution $F_{Q_1|E_1=1,A=a}$ and $F_{Q_2^{q_1=0}|E_2^{q_1=0}=1,A=a}$.

Even if there is no population-level waning, that is, if

$$E_G[\Delta Y_1^{a=1,q_1=q}] = E_G[\Delta Y_2^{a=1,q_1=0,q_2=q}]$$

$$E_G[\Delta Y_1^{a=0,q_1=q}] = E_G[\Delta Y_2^{a=0,q_1=0,q_2=q}]$$

with probability 1, $\forall q \in \mathcal{Q}$, holds, the left hand sides of Equations (9)-(10) are not necessarily equal. If the distribution of intensities changes from time 1 to time 2, for example, because a new strain enters the population during follow-up, with a different infectivity, then the expectations taken with respect to the conditional distributions $F_{Q_1|E_1=1,A=a}$ and $F_{Q_2^{q_1=0}|E_2^{q_1=0}=1,A=a}$ need not coincide.

Thus, assume the following, stated first in Janvin and Stensrud [1]:

Assumption 11 (Stationarity of exposure versions among the exposed)**.**

$$P_G(Q_1 \leq q|E_1 = 1, A = a) = P_G(Q_2^{q_1=0} \leq q|E_2^{q_1=0}, A = a) \ w.p. \ 1.$$

Assumption 11 allows for different distribution of exposures (E_1, E_2) between the intervals, however, it requires that, conditional on being exposed in a given interval, the distribution of the "strength of the exposure" is the same in the two.

With a slight reformulation of Proposition 3 of Janvin and Stensrud [1] we can state the following:

Proposition 5 (Restatement of Proposition 3 in Janvin and Stensrud [1]). *Under Assumptions 1,3, 12 (i), (ii), 13, 14, and 11 from our manuscript and Assumptions 4, 9-12 from Janvin and Stensrud [1], the null hypothesis*

$$E_G[\Delta Y_1^{a=1,q_1=q}] = E_G[\Delta Y_2^{a=1,q_1=0,q_2=q}]$$

$$E_G[\Delta Y_1^{a=0,q_1=q}] = E_G[\Delta Y_2^{a=0,q_1=0,q_2=q}]$$

for all $q \in \mathcal{Q}$ implies

$$\frac{E[\Delta Y_2|A=1]}{E[\Delta Y_1 + \Delta Y_2|A=0]} \leq \frac{E[\Delta Y_1|A=1]}{E[\Delta Y_1|A=0]} \leq \frac{E[\Delta Y_1 + \Delta Y_2|A=1]}{E[\Delta Y_2|A=0]} \tag{11}$$

See proof in Appendix D of Janvin and Stensrud [1].

Thus, two one-sided tests based on observed quantities can be constructed for the null hypothesis of no waning, whether the confidence interval for the difference between $\frac{E[\Delta Y_1|A=1]}{E[\Delta Y_1|A=0]}$ and the corresponding bounds excludes 0. However, the rejection of the null hypothesis implies only that there exists at least one intensity $q \in \mathcal{Q}$ for which the expected potential outcome changed over time, but we cannot identify, the subset $\mathcal{Q}' \subseteq \mathcal{Q}$, for which the vaccine has waned.

Our null hypothesis, Hypothesis 0 is contingent on binary exposure intensity: an individual is either exposed or not. While waning under multiple versions of exposures can

still be tested, possibly wide bounds

$$\left[\frac{E[\Delta Y_2|A=1]}{E[\Delta Y_1+\Delta Y_2|A=0]}, \frac{E[\Delta Y_1+\Delta Y_2|A=1]}{E[\Delta Y_2|A=0]}\right]$$

come at the cost of substantial loss of power. Therefore, the originally proposed test can be interpreted as a joint hypothesis of no waning and an all or nothing vaccine effect under exposure. When the original test of the equality of incidence ratios is rejected, this may reflect either waning vaccine effects or "leaky" vaccines [8], in which framework, the expected potential outcome under exposure is sensitive to exposure intensity.

The reason why, in settings with multiple exposure intensities, not even a sharp null hypothesis of no waning yields a valid test based on the equality of incidence ratios is that the joint distribution of exposure intensities (Q_1, Q_2) and susceptibility (U_Y) is unknown. It is possible that some individuals exposed at time 1 had underlying susceptibility such that $\Delta Y_1^{a,q_1=q} = 1$ for any $q > 0$. Had they been isolated and then exposed at time 2, they would develop the outcome regardless of exposure intensity, even if no waning occurred. By contrast, among those who developed the outcome at time 1, only because the exposure intensity exceeded some threshold q^*, had they been isolated in the first interval, the expected outcome under exposure at time 2 can be expressed as a function of the intensity distributions and q^*, using the stationarity Assumption 11. We conjecture that by explicitly modelling the joint distribution of susceptibility and exposure intensity, under the assumptions of Proposition 5, tighter bounds can be derived for the time 2 challenge effect, thereby increasing the power of our test. Building on results on vaccine effects in the presence of competing strains [9], this direction will be the focus of follow-up work.

S4 Principal Strata at Different Times

Suppose that we are conducting a 4-arm trial, as described in Section 1 and depicted in Figure 2 of the main manuscript. Consider a counterfactual causal model such that there exists a pre-treatment variable, denoted by $T \in \{1, \ldots, 16\}$, such that

$$T = 1 \implies (\Delta Y_1^{a=1,e_1=1} = 0, \Delta Y_1^{a=0,e_1=1} = 0, \Delta Y_2^{a=1,e_1=0,e_2=1} = 0, \Delta Y_2^{a=0,e_1=0,e_2=1} = 0)$$

$$T = 2 \implies (\Delta Y_1^{a=1,e_1=1} = 0, \Delta Y_1^{a=0,e_1=1} = 0, \Delta Y_2^{a=1,e_1=0,e_2=1} = 0, \Delta Y_2^{a=0,e_1=0,e_2=1} = 1)$$

$$T = 3 \implies (\Delta Y_1^{a=1,e_1=1} = 0, \Delta Y_1^{a=0,e_1=1} = 0, \Delta Y_2^{a=1,e_1=0,e_2=1} = 1, \Delta Y_2^{a=0,e_1=0,e_2=1} = 0)$$

$$T = 4 \implies (\Delta Y_1^{a=1,e_1=1} = 0, \Delta Y_1^{a=0,e_1=1} = 0, \Delta Y_2^{a=1,e_1=0,e_2=1} = 1, \Delta Y_2^{a=0,e_1=0,e_2=1} = 1)$$

$$T = 5 \implies (\Delta Y_1^{a=1,e_1=1} = 0, \Delta Y_1^{a=0,e_1=1} = 1, \Delta Y_2^{a=1,e_1=0,e_2=1} = 0, \Delta Y_2^{a=0,e_1=0,e_2=1} = 0)$$

$$T = 6 \implies (\Delta Y_1^{a=1,e_1=1} = 0, \Delta Y_1^{a=0,e_1=1} = 1, \Delta Y_2^{a=1,e_1=0,e_2=1} = 0, \Delta Y_2^{a=0,e_1=0,e_2=1} = 1)$$

$$T = 7 \implies (\Delta Y_1^{a=1,e_1=1} = 0, \Delta Y_1^{a=0,e_1=1} = 1, \Delta Y_2^{a=1,e_1=0,e_2=1} = 1, \Delta Y_2^{a=0,e_1=0,e_2=1} = 0)$$

$$T = 8 \implies (\Delta Y_1^{a=1,e_1=1} = 0, \Delta Y_1^{a=0,e_1=1} = 1, \Delta Y_2^{a=1,e_1=0,e_2=1} = 1, \Delta Y_2^{a=0,e_1=0,e_2=1} = 1)$$

$$T = 9 \implies (\Delta Y_1^{a=1,e_1=1} = 1, \Delta Y_1^{a=0,e_1=1} = 0, \Delta Y_2^{a=1,e_1=0,e_2=1} = 0, \Delta Y_2^{a=0,e_1=0,e_2=1} = 0)$$

$$T = 10 \implies (\Delta Y_1^{a=1,e_1=1} = 1, \Delta Y_1^{a=0,e_1=1} = 0, \Delta Y_2^{a=1,e_1=0,e_2=1} = 0, \Delta Y_2^{a=0,e_1=0,e_2=1} = 1)$$

$$T = 11 \implies (\Delta Y_1^{a=1,e_1=1} = 1, \Delta Y_1^{a=0,e_1=1} = 0, \Delta Y_2^{a=1,e_1=0,e_2=1} = 1, \Delta Y_2^{a=0,e_1=0,e_2=1} = 0)$$

$$T = 12 \implies (\Delta Y_1^{a=1,e_1=1} = 1, \Delta Y_1^{a=0,e_1=1} = 0, \Delta Y_2^{a=1,e_1=0,e_2=1} = 1, \Delta Y_2^{a=0,e_1=0,e_2=1} = 1)$$

$$T = 13 \implies (\Delta Y_1^{a=1,e_1=1} = 1, \Delta Y_1^{a=0,e_1=1} = 1, \Delta Y_2^{a=1,e_1=0,e_2=1} = 0, \Delta Y_2^{a=0,e_1=0,e_2=1} = 0)$$

$$T = 14 \implies (\Delta Y_1^{a=1,e_1=1} = 1, \Delta Y_1^{a=0,e_1=1} = 1, \Delta Y_2^{a=1,e_1=0,e_2=1} = 0, \Delta Y_2^{a=0,e_1=0,e_2=1} = 1)$$

$$T = 15 \implies (\Delta Y_1^{a=1,e_1=1} = 1, \Delta Y_1^{a=0,e_1=1} = 1, \Delta Y_2^{a=1,e_1=0,e_2=1} = 1, \Delta Y_2^{a=0,e_1=0,e_2=1} = 0)$$

$$T = 16 \implies (\Delta Y_1^{a=1,e_1=1} = 1, \Delta Y_1^{a=0,e_1=1} = 1, \Delta Y_2^{a=1,e_1=0,e_2=1} = 1, \Delta Y_2^{a=0,e_1=0,e_2=1} = 1)$$

The counterfactual groups above are a composite of the 4 conventional deterministic

principal strata defined at each point in time. For example, someone may be doomed at time 1, while immune at time 2, which corresponds to $T = 4$. The transition from one principal stratum to another corresponds to a waning at the individual level, as one's response to exposure changes, at least under one of the two treatment levels.

In this general setting, the challenge effects are

$$\begin{aligned} VE_1^{\text{challenge}} &= \frac{P\big(T \in \{9, \ldots, 16\}\big)}{P\big(T \in \{5, \ldots, 8, 13, \ldots, 16\}\big)} \\ VE_2^{\text{challenge}} &= \frac{P\big(T \in \{3, 4, 7, 8, 11, 12, 15, 16\}\big)}{P\big(T \in \{2, 4, 6, 8, 10, 12, 14, 16\}\big)}, \end{aligned}$$

which are equal to the observed incidence ratios under a suitable set of assumptions, as shown in Appendix S6.

Corollary 1 (Principal Strata under the null)**.** *Suppose Hypothesis 0 holds. Then*

$$P(T \in \{1, 6, 11, 16\}) = 1$$

Proof. Under the null hypothesis, and by partitioning

$$\begin{aligned} 0 &\overset{H_0}{=} P(\Delta Y_1^{a=1,e_1=1} = 0, \Delta Y_2^{a=1,e_1=0,e_2=1} = 1) \\ &= P(\Delta Y_1^{a=1,e_1=1} = 0, \Delta Y_1^{a=0,e_1=1} = 0, \Delta Y_2^{a=1,e_1=0,e_2=1} = 1, \Delta Y_2^{a=0,e_1=0,e_2=1} = 0) \\ &+ P(\Delta Y_1^{a=1,e_1=1} = 0, \Delta Y_1^{a=0,e_1=1} = 0, \Delta Y_2^{a=1,e_1=0,e_2=1} = 1, \Delta Y_2^{a=0,e_1=0,e_2=1} = 1) \\ &+ P(\Delta Y_1^{a=1,e_1=1} = 0, \Delta Y_1^{a=0,e_1=1} = 1, \Delta Y_2^{a=1,e_1=0,e_2=1} = 1, \Delta Y_2^{a=0,e_1=0,e_2=1} = 0) \\ &+ P(\Delta Y_1^{a=1,e_1=1} = 0, \Delta Y_1^{a=0,e_1=1} = 1, \Delta Y_2^{a=1,e_1=0,e_2=1} = 1, \Delta Y_2^{a=0,e_1=0,e_2=1} = 1). \end{aligned}$$

Therefore, by the axioms of probability $P(T = 3) = P(T = 4) = P(T = 7) = P(T = 8) = 0$. Similarly, under the null

$$\begin{aligned} &P(\Delta Y_1^{a=1,e_1=1} = 1, \Delta Y_2^{a=0,e_1=0,e_2=1} = 0) = P(\Delta Y_1^{a=0,e_1=1} = 0, \Delta Y_2^{a=0,e_1=0,e_2=1} = 1) \\ &= P(\Delta Y_1^{a=0,e_1=1} = 1, \Delta Y_2^{a=0,e_1=0,e_2=1} = 0) = 0, \end{aligned}$$

which implies that

$$P(T=9)=P(T=10)=P(T=13)=P(T=14)=0$$

$$P(T=2)=P(T=4)=P(T=10)=P(T=12)=0$$

$$P(T=5)=P(T=7)=P(T=13)=P(T=15)=0.$$

Thus, we have that $P(T\in\{2,3,4,5,7,8,9,10,12,13,14,15\})=0$, which means that $P(T\in\{1,6,11,16\})=1$. $\square$

It follows that, under the null hypothesis, the challenge effects are equal to

$$VE_1^{\text{challenge}}=\frac{P\big(T\in\{9,\dots,16\}\big)}{P\big(T\in\{5,\dots,8,13,\dots,16\}\big)}\overset{H_0}{=}\frac{P\big(T\in\{11,16\}\big)}{P\big(T\in\{6,16\}\big)}$$
$$VE_2^{\text{challenge}}=\frac{P\big(T\in\{3,4,7,8,11,12,15,16\}\big)}{P\big(T\in\{2,4,6,8,10,12,14,16\}\big)}\overset{H_0}{=}\frac{P\big(T\in\{11,16\}\big)}{P\big(T\in\{6,16\}\big)},$$

which shows their equality under the null.

We argue for the equivalence of the population level and the sharp no waning, under the sharp no waning of the placebo, that is, $\Delta Y_1^{a=0,e_1=1}=\Delta Y_2^{a=0,e_1=0,e_2=1}$ with probability 1. We believe this is a plausible assumption, as the effect of the placebo should not change over time for an individual if they are isolated; hence, their immune system is not challenged before the exposure. Therefore, if they were to develop the outcome upon exposure at time 1, then they would develop it at time 2, given that the exposures are identical. Similarly, if they were to remain event-free at time 1 due to natural immunity, for example, due to genetic reasons, a quality that would not change over time and they would remain event-free at time 2 as well.

By identical arguments as in the proof above, the sharp no waning of the placebo implies that

$$P(T\in\{2,4,5,7,10,12,13,15\})=0.$$

In this setting, the challenge effects are

$$VE_1^{\text{challenge}} = \frac{P\big(T \in \{9,\dots,16\}\big)}{P\big(T \in \{5,\dots,8,13,\dots,16\}\big)} = \frac{P\big(T \in \{9,11,14,16\}\big)}{P\big(T \in \{6,8,14,16\}\big)}$$

$$VE_2^{\text{challenge}} = \frac{P\big(T \in \{3,4,7,8,11,12,15,16\}\big)}{P\big(T \in \{2,4,6,8,10,12,14,16\}\big)} = \frac{P\big(T \in \{3,8,11,16\}\big)}{P\big(T \in \{6,8,14,16\}\big)}.$$

Therefore, $VE_1^{\text{challenge}} = VE_2^{\text{challenge}}$, when the null hypothesis does not hold, if and only if $\big(T \in \{9,11,14,16\}\big) = \big(T \in \{3,8,11,16\}\big)$, that is equivalent to

$$P(T = 9) + P(T = 14) = P(T = 3) + P(T = 8).$$

Here, $T = 9$ corresponds to individuals who are harmed by vaccination in the first interval but who would have been immune at time 2 had they instead been isolated in prior to that. In contrast, $T = 14$ denotes individuals who are doomed in the first interval but who would have been helped in the second interval under following isolation. Thus, $T = 9 \cup T = 14$ comprises those whose potential outcome under vaccination improves from time 1 to time 2.

Similarly, $T = 3 \cup T = 8$ comprises individuals who transition from immune to harmed or from helped to doomed, respectively; that is, those whose potential outcome under vaccination worsens from time 1 to time 2. Informally, the equality above implies that, in the vaccinated arm, the number of individuals experiencing *positive* waning is equal to the number experiencing *negative* waning.

In general, it is unlikely that the vaccine effect improves over time, i.e., $P(T \in \{9,14\}) \approx 0$. Even if such improvement were possible, an exact balance between vaccine improvement and deterioration would be even less plausible. Therefore, the null hypothesis of no individual-level waning is plausibly equivalent to the null hypothesis of no population-level waning.

S5 On the Assumptions of Janvin and Stensrud (2024)

Similar to Janvin and Stensrud [1], because we consider data from a randomized controlled trial, we take the following three conventional assumptions for granted:

Assumption 12 (Consistency)**.** Suppose that the interventions on the treatment A, exposures E_1, E_2, and the first timepoint survival $\Delta Y_1 = 0$ are well-defined. That is

(i) if $A = a$ then $E_1 = E_1^a, \Delta Y_1 = \Delta Y_1^a, E_2 = E_2^a, \Delta Y_2 = \Delta Y_2^a$,

(ii) if $E_1^a = e_1$ then $\Delta Y_1^a = \Delta Y_1^{a,e_1}, E_2^a = E_2^{a,e_1}, \Delta Y_2^a = \Delta Y_2^{a,e_1}$,

(iii) if $\Delta Y_1^{a,e_1} = 0$ then $E_2^{a,e_1} = E_2^{a,e_1,\delta y_1=0}, \Delta Y_2^{a,e_1} = \Delta Y_2^{a,e_1,\delta y_1=0}$,

(iv) if $E_2^{a,e_1,\delta y_1=0} = e_2$ then $\Delta Y_2^{a,e_1,\delta y_1=0} = \Delta Y_2^{a,e_1,\delta y_1=0,e_2}$,

(v) if $E_2^{a,e_1=0} = e_2$ then $\Delta Y_2^{a,e_1=0} = \Delta Y_2^{a,e_1=0,e_2}$,

for all $a, e_1, e_2 \in \{0, 1\}$.

Assumption 13 (Treatment exchangeability)**.**

$$E_1^a, \Delta Y_1^a, \Delta Y_1^{a,e_1}, E_2^{a,e_1}, E_2^{a,e_1\delta y_1=0}, \Delta Y_2^{a,e_1,\delta y_1=0,e_2} \perp\!\!\!\perp A$$

for all $a, e_1, e_2 \in \{0, 1\}$.

Assumption 14 (Positivity)**.**

$$P(A = a) > 0 \text{ for all } a \in \{0, 1\}.$$

Assumptions 12-14 are close to the RCT assumptions set by Janvin and Stensrud [1]. The most important distinction is that we suppose it is possible to intervene on ΔY_1, with a well-defined intervention; thus, consistency holds if $\Delta Y_1 = 0$. Accordingly, we state

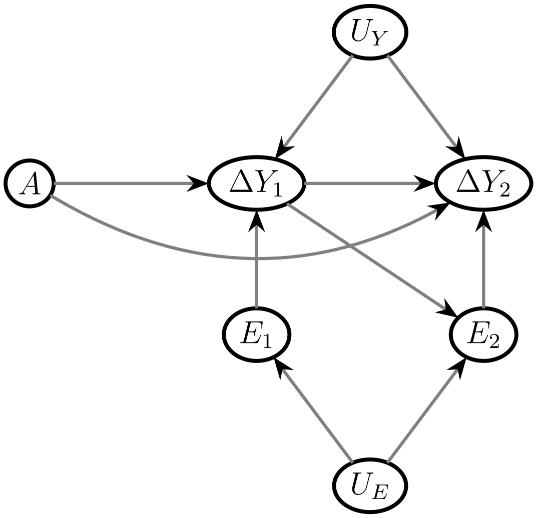

Figure S2: Directed acyclic graph illustrating the observed data structure.

the treatment exchangeability assumption for potential outcomes under the intervention $\delta y_1 = 0$.

Assumption 1 states that in order to develop the outcome at a given timepoint, exposure at the same timepoint should have happened. The version stated in the main document is stronger than the one proposed by Janvin and Stensrud [1]: Assumption 1 needs to hold even if there was no intervention at time 1 isolating the individuals. That is, there can be no causal effect of time 1 exposure at the time 2 outcome, that is not mediated through ΔY_1 or E_2, which is also a sufficient condition for Assumption 2.

While Assumption 13 already imposed structural constraints on the relationship of the variables, we can take those for granted by the randomized design. Assumptions 2-4 further restrict the dependencies between the variables. Janvin and Stensrud [1] argues for a plausible structure of the observed data, depicted in Figure S2. The casual DAG satisfies Assumption 2-4 and 5.

Assumption 2 is not stated explicitly by Janvin and Stensrud [1], but, the same argument is used for their Assumption 4: no causal path is expected to exist from E_1 to ΔY_2

that is not through ΔY_1 (we also allow mediation through E_2). However, if there exists such a path under an intervention $\delta y_1 = 0$, Assumption 2 is violated, while Assumption 4 of Janvin and Stensrud [1] can still hold, even though this occurs in a contrived scenario.

Assumption 2 facilitates our discussion and it is conceivable in many scenarios, for example antiretroviral post-exposure prophylaxis (PEP) treatment following exposure to HIV, or defining exposure intervention as spraying infectious particles of the respiratory disease towards the patient, however ensuring survival by an intervention instructing them to wear medical face shields. However, Assumption 2 is not essential for our results to hold, and we can avoid using potential outcomes under interventions on survival.

Assumption 15.

$$E[\Delta Y_2^{a,e_1=1,e_2=1} | \Delta Y_1^{a,e_1=1} = 0] = E[\Delta Y_2^{a,e_1=0,e_2=1} | \Delta Y_1^{a,e_1=1} = 0]$$

Assumption 15 posits that, among individuals who survived time 1, despite exposure, the expected event rate at time 2 is the same following isolation as it is following exposure. Although Assumption 15 is cross-world, it is plausible when E_1 has no direct effect on ΔY_2. In that case the effect of E_1 is entirely mediated through time 1 survival. Accordingly in models such as NPSEM-IE-s [10], conditioning on survival makes the expected time 2 outcome invariant to interventions on E_1.

Under the null hypothesis, $E[\Delta Y_2^{a,e_1=0,e_2=1} | \Delta Y_1^{a,e_1=1} = 0] = 0$, thus under Assumption 15 $E[\Delta Y_2^{a,e_1=1,e_2=1} | \Delta Y_1^{a,e_1=1} = 0] = 0$, thus Equation (13) equals to 0, which shows that $E[\Delta Y_2^a | E_1^a = 1] P(E_1^a = 1) = 0$, and proves our result.

Assumption 3 is equivalent to Assumption 3 of Janvin and Stensrud [1].

Finally, under Assumption 13, Assumption 4 is equivalent to Assumption 4 of Janvin and Stensrud [1].

Therefore, to test the null hypothesis, Assumption 1 and 2 slightly restrict their counterparts from Janvin and Stensrud [1]. However, no other independencies are imposed that would not hold under the DAG proposed to represent a plausible data-generating mechanism. While all of the above discussed assumptions hold under the DAG in Figure S2, they would hold even if E_1 had a direct effect on E_2, thus in this sense, our conditions are more general than the ones imposed by Janvin and Stensrud [1].

S6 Proof of Proposition 1

Proof. The observed incidence rate ratio at time 1 is equal to

$$
\begin{aligned}
1 - VE_1^{\text{challenge}} &:= \frac{E[\Delta Y_1^{a=1,e_1=1}]}{E[\Delta Y_1^{a=0,e_1=1}]} \\
&\overset{\text{Assumption 4}}{=} \frac{E[\Delta Y_1^{a=1,e_1=1} | E_1^{a=1} = 1]}{E[\Delta Y_1^{a=0,e_1=1} | E_1^{a=0} = 1]} \\
&\overset{\text{Assumption 12}}{=} \frac{E[\Delta Y_1^{a=1} | E_1^{a=1} = 1]}{E[\Delta Y_1^{a=0} | E_1^{a=0} = 1]} \\
&\overset{\text{Assumption 1}}{=} \frac{E[\Delta Y_1^{a=1}]/P(E_1^{a=1} = 1)}{E[\Delta Y_1^{a=0}]/P(E_1^{a=0} = 1)} \\
&\overset{\text{Assumption 3}}{=} \frac{E[\Delta Y_1^{a=1}]}{E[\Delta Y_1^{a=0}]} \\
&\overset{\text{Assumption 13, 14}}{=} \frac{E[\Delta Y_1^{a=1} | A = 1]}{E[\Delta Y_1^{a=0} | A = 0]} \\
&\overset{\text{Assumption 12}}{=} \frac{E[\Delta Y_1 | A = 1]}{E[\Delta Y_1 | A = 0]}
\end{aligned}
$$

At time 2, the incidence rate for the arm $A = a$ is

$$
\begin{aligned}
E[\Delta Y_2|A=a] &\overset{\text{Assumption 12, 13, 14}}{=} E[\Delta Y_2^a] \\
&= E[\Delta Y_2^a|E_1^a=0]P(E_1^a=0) \\
&+ E[\Delta Y_2^a|E_1^a=1]P(E_1^a=1).
\end{aligned}
$$

Considering the first term

$$
\begin{aligned}
E[\Delta Y_2^a|E_1^a=0]P(E_1^a=0) &\overset{\text{Assumption 1}}{=} E[\Delta Y_2^a|E_1^a=0, E_2^a=1]P(E_2^a=1|E_1^a=0)P(E_1^a=0) \\
&\overset{\text{Assumption 12}}{=} E[\Delta Y_2^{a,e_1=0}|E_1^a=0, E_2^{a,e_1=0}=1]P(E_2^{a,e_1=0}=1|E_1^a=0)P(E_1^a=0) \\
&\overset{\text{Assumption 4}}{=} E[\Delta Y_2^{a,e_1=0}|E_2^{a,e_1=0}=1]P(E_2^{a,e_1=0}=1|E_1^a=0)P(E_1^a=0) \\
&\overset{\text{Assumption 12}}{=} E[\Delta Y_2^{a,e_1=0,e_2=1}|E_2^{a,e_1=0}=1]P(E_2^{a,e_1=0}=1, E_1^a=0) \\
&\overset{\text{Assumption 4}}{=} E[\Delta Y_2^{a,e_1=0,e_2=1}]P(E_2^{a,e_1=0}=1, E_1^a=0).
\end{aligned}
$$

The second term equals

$$
\begin{aligned}
E[\Delta Y_2^a|E_1^a=1]P(E_1^a=1) &= E[\Delta Y_2^a|E_1^a=1, \Delta Y_1^a=0]P(\Delta Y_1^a=0|E_1^a=1)P(E_1^a=1) \\
&\overset{\text{Assumption 1}}{=} E[\Delta Y_2^a|E_1^a=1, \Delta Y_1^a=0, E_2^a=1] \\
&\times P(E_2^a=1|E_1^a=1, \Delta Y_1^a=0)P(\Delta Y_1^a=0|E_1^a=1)P(E_1^a=1) \\
&\overset{\text{Assumption 12}}{=} E[\Delta Y_2^{a,e_1=1}|E_1^a=1, \Delta Y_1^{a,e_1=1}=0, E_2^{a,e_1=1}=1] \\
&\times P(E_2^{a,e_1=1}=1|E_1^a=1, \Delta Y_1^{a,e_1=1}=0)P(\Delta Y_1^{a,e_1=1}=0|E_1^a=1)P(E_1^a=1)
\end{aligned}
\tag{12}
$$

Using Assumption 12

$$
E[\Delta Y_2^{a,e_1=1}|E_1^a=1, \Delta Y_1^{a,e_1=1}=0, E_2^{a,e_1=1}=1] \tag{13}
$$

$$
= E[\Delta Y_2^{a,e_1=1,\delta y_1=0,e_2=1}|E_1^a=1, \Delta Y_1^{a,e_1=1}=0, E_2^{a,e_1=1,\delta y_1=0}=1]. \tag{14}
$$

As shown in Remark 1, under Assumption 2 and the null hypothesis, $\Delta Y_2^{a,e_1=1,\delta y_1=0,e_2=1} = \Delta Y_1^{a,e_1=1}$. Therefore

$$E[\Delta Y_2^{a,e_1=1,\delta y_1=0,e_2=1}|E_1^a = 1, \Delta Y_1^{a,e_1=1} = 0, E_2^{a,e_1=1,\delta y_1=0} = 1] = 0,$$

which shows that $E[\Delta Y_2^a|E_1^a = 1]P(E_1^a = 1) = 0$. Finally,

$$\begin{aligned}
\frac{E[\Delta Y_2|A=1]}{E[\Delta Y_2|A=1]} &= \frac{E[\Delta Y_2^{a=1}|E_1^{a=1}=0]P(E_1^{a=1}=0)}{E[\Delta Y_2^{a=0}|E_1^{a=0}=0]P(E_1^{a=0}=0)} \\
&= \frac{E[\Delta Y_2^{a=1,e_1=0,e_2=1}]P(E_2^{a=1,e_1=0}=1, E_1^{a=1}=0)}{E[\Delta Y_2^{a=0,e_1=0,e_2=1}]P(E_2^{a=0,e_1=0}=1, E_1^{a=0}=0)} \\
&\overset{\text{Assumption 3}}{=} \frac{E[\Delta Y_2^{a=1,e_1=0,e_2=1}]}{E[\Delta Y_2^{a=0,e_1=0,e_2=1}]} := 1 - VE_2^{\text{challenge}}
\end{aligned}$$

Under the null hypothesis $E[\Delta Y_1^{a,e_1=1}] = E[\Delta Y_2^{a,e_1=0,e_2=1}]$, which implies $VE_1^{\text{challenge}} = VE_2^{\text{challenge}}$, and thus the equality $IR_1 = IR_2$ holds. □

S7 Interpretation of the p-value under parametric assumptions

We introduced a test for waning that is valid under non-parametric assumptions and is agnostic to the exact form of the data-generating mechanism. Under these assumptions, while the p-value is informative about the existence of waning, it is not necessarily informative about its magnitude, that is, a lower p-value does not necessarily imply a larger discrepancy between the time 1 and time 2 challenge effects. However, if the mechanism underlying waning is specified, for example, by defining waning as a particular transition from one principal stratum to another, then stronger claims can be made about the magnitude of waning.

Similarly to Appendix S9 consider the following data-generating mechanism:

$$
\begin{aligned}
U_Y &\sim F_{U_Y} \\
U_E &\sim F_{U_E} \\
A &:= \text{Bernoulli}(\pi) \\
T_1|U_Y &:= \text{Categorical}(p_{doomed_1}(U_Y), p_{helped_1}(U_Y), p_{harmed_1}(U_Y), p_{immune_1}(U_Y)) \\
W|U_Y, T_1 &:= \begin{cases} \text{Bernoulli}(\omega(U_Y)), & T_1 = \text{helped}, \\ 0, & T_1 \neq \text{helped}. \end{cases} \\
T_2|T_1, W &:= \begin{cases} \text{doomed}, & T_1 = \text{doomed}, \\ \text{doomed}, & T_1 = \text{helped},\ W = 1, \\ \text{helped}, & T_1 = \text{helped},\ W = 0, \\ \text{harmed}, & T_1 = \text{harmed}, \\ \text{immune}, & T_1 = \text{immune}. \end{cases} \\
E_1|U_E &:= \text{Bernoulli}(p_{E_1}(U_E)) \\
\Delta Y_1 &:= E_1 \times \big(I(T_1 = \text{doomed}) + (1 - A) \times I(T_1 = \text{helped}) + A \times I(T_1 = \text{harmed})\big) \\
E_2|U_E, \Delta Y_1 &:= \text{Bernoulli}(p_{E_2}(U_E, \Delta Y_1)) \\
\Delta Y_2 &:= (1 - \Delta Y_1) \times E_2 \times \big(I(T_2 = \text{doomed}) + (1 - A) \times I(T_2 = \text{helped}) + A \times I(T_2 = \text{harmed})\big).
\end{aligned}
$$

In the proposed data-generating mechanism, we assumed that the only possible transition between principal strata over time is from *helped* to *doomed*. This transition corresponds to a decrease in vaccine efficacy, while the placebo response is assumed not to wane. Therefore, individual-level vaccine improvement is ruled out by construction, which is a strong and

possibly unrealistic assumption. This can be interpreted as a monotonicity assumption over time, requiring that susceptibility under treatment can only increase, not decrease.

For ease of notation, let $S_{a,k}$ denote the indicator that an individual would develop the outcome at time k under treatment a and exposure, had they remained event-free up to that time. For example, $S_{1,1} = 1$ indicates that the individual belongs to $T_1 \in \{\text{doomed}, \text{harmed}\}$, whereas $S_{0,2} = 1$ indicates that the individual belongs to $T_2 \in \{\text{doomed}, \text{helped}\}$. In this notation, we have

$$\begin{aligned}
1 - VE_1^{\text{challenge}} &= \frac{P(S_{1,1} = 1)}{P(S_{0,1} = 1)} = \frac{P(T_1 \in \{\text{doomed, harmed}\})}{P(T_1 \in \{\text{doomed, helped}\})}, \\
1 - VE_2^{\text{challenge}} &= \frac{P(S_{1,2} = 1)}{P(S_{0,2} = 1)} = \frac{P(T_2 \in \{\text{doomed, harmed}\})}{P(T_2 \in \{\text{doomed, helped}\})}.
\end{aligned}$$

In contrast, the incidence ratios are

$$\begin{aligned}
IR_1 &= \frac{P(E_1 = 1, S_{1,1} = 1)}{P(E_1 = 1, S_{0,1} = 1)}, \\
IR_2 &= \frac{P(E_2 = 1, \Delta Y_1 = 0, S_{1,2} = 1)}{P(E_2 = 1, \Delta Y_1 = 0, S_{0,2} = 1)}.
\end{aligned}$$

Since in the specified data-generating mechanism no waning of the placebo holds, $S_{0,1} = S_{0,2}$. Moreover, because the only possible transition between principal strata is from *helped* to *doomed*, we have $S_{1,1} \leq S_{1,2}$

Since the principal strata, T_1, is independent of the exposure E_1, similarly to Proposition 1, we obtain

$$IR_1 = \frac{P(E_1 = 1, S_{1,1} = 1)}{P(E_1 = 1, S_{0,1} = 1)} = \frac{P(S_{1,1} = 1)}{P(S_{0,1} = 1)} = 1 - VE_1^{\text{challenge}}.$$

To rewrite IR_2, we consider the two arms separately. Using the notation for susceptibility,

we can rewrite

$$E[\Delta Y_2] = \begin{cases} E[(1-\Delta Y_1)E_2 S_{1,2}] = E[(1-E_1 S_{1,1})E_2 S_{1,2}] \text{ if } A=1, \\ \\ E[(1-\Delta Y_1)E_2 S_{0,2}] = E[(1-E_1 S_{0,1})E_2 S_{0,2}] \text{ if } A=0. \end{cases}$$

Since no waning of the placebo holds, $(1-E_1 S_{0,1})S_{0,2} = (1-E_1)S_{0,1}$, thus

$$E[\Delta Y_2 | A=0] = E[(1-E_1)p_{E_2}(0,U_E)]P(S_{0,1}=1),$$

where we further used that we are only interested in the time 2 exposure of the survivors, thus making the time 2 exposure independent of the time 1 susceptibility.

For the treated, $S_{1,2}=1$ can occur in two disjoint ways: either the individual was already susceptible at time 1, $S_{1,1}=1$, or the individual becomes newly susceptible due to waning, $\{S_{1,1}=0, W=1, S_{1,2}\} = \{T_1 = \text{helped}, W=1\}$. The first group would develop the outcome at time 1, if they were exposed, hence, they contribute to the time 2 events only if they were unexposed at time 1 and then exposed at time 2, which happens with probability $E[(1-E_1)p_{E_2}(0,U_E)]$.

In the second group, individuals remain event-free at time 1, regardless of exposure, thus only the time 2 exposure needs to be considered, which occurs with probability $E[p_{E_2}(0,U_E)]$. Putting these two groups together, the observed time 2 event rate under treatment is

$$\begin{aligned} E[\Delta Y_2 | A=1] = E[(1-E_1)p_{E_2}(0,U_E)]P(S_{1,1}=1) \\ + E[p_{E_2}(0,U_E)]P(S_{1,1}=0, W=1, S_{1,2}=1), \end{aligned}$$

thus IR_2 is

$$IR_2 = \frac{E[(1-E_1)p_{E_2}(0,U_E)]P(S_{1,1}=1) + E[p_{E_2}(0,U_E)]P(S_{1,1}=0, W=1, S_{1,2}=1)}{E[(1-E_1)p_{E_2}(0,U_E)]P(S_{0,1}=1)}.$$

Our test statistic is $\log(\widehat{IR}_2) - \log(\widehat{IR}_1) = \log\left(\frac{\widehat{IR}_2}{\widehat{IR}_1}\right)$. Under the proposed data-generating mechanism, the ratio of the incidence ratios is equal to

$$\frac{IR_2}{IR_1} = \frac{E[(1-E_1)p_{E_2}(0,U_E)]P(S_{1,1}=1) + E[p_{E_2}(0,U_E)]P(S_{1,1}=0, W=1, S_{1,2}=1)}{E[(1-E_1)p_{E_2}(0,U_E)]P(S_{1,1}=1)}$$
$$= 1 + \frac{E[p_{E_2}(0,U_E)]}{E[(1-E_1)p_{E_2}(0,U_E)]}\frac{P(S_{1,1}=0, W=1, S_{1,2}=1)}{P(S_{1,1}=1)}.$$

However, $P(S_{1,1}=0, W=1, S_{1,2}=1)$ is precisely the proportion of individuals for whom waning has occurred, that is, the extent of waning. By analogous rewriting, we can show that

$$\frac{1-VE_2^{\text{challenge}}}{1-VE_1^{\text{challenge}}} = 1 + \frac{P(S_{1,1}=0, W=1, S_{1,2}=1)}{P(S_{0,1}=1)}.$$

Therefore, provided that the probability of exposure among survivors at time 2 is non-zero, the test statistic is strictly increasing in the waning term. Equivalently, as the ratio $\frac{1-VE_2^{\text{challenge}}}{1-VE_1^{\text{challenge}}}$ increases, the test statistic increases as well.

In conclusion, under the proposed parametrized version of waning, the test statistic and the corresponding p-value are informative about the extent of waning. Thus, under suitable modeling assumptions, the test can be used not only to detect waning but also to quantify it.

S8 Hazard-ratio based waning under the null hypothesis

Suppose that, similarly to Appendix S9, the variables are distributed according to the following data-generating mechanism:

$$A \sim P_A \tag{15}$$

$$T \sim \text{Categorical}\{p_{doomed}, p_{helped}, p_{harmed}, p_{immune}\} \tag{16}$$

$$E_1 \sim P_{E_1} \tag{17}$$

$$\Delta Y_1 = E_1 \times \big(I(T_1 = 1) + (1 - A) \times I(T = 2) + A \times I(T = 3)\big) \tag{18}$$

$$E_2 = (1 - \Delta Y_1) \times P_{E_2} \tag{19}$$

$$\Delta Y_2 = E_2 \times \big(I(T_1 = 1) + (1 - A) \times I(T = 2) + A \times I(T = 3)\big) \tag{20}$$

For simplicity, consider $P_A \sim \text{Bernoulli}(0.5)$, and $P_{E_1} \sim \text{Bernoulli}(p_{E_1})$, $P_{E_2} \sim \text{Bernoulli}(p_{E_2})$. Since at randomization all individuals are event-free, it follows that

$$IR_1 = HR_1 \overset{H_0}{=} \frac{P\big(T \in \{11, 16\}\big)}{P\big(T \in \{6, 16\}\big)} = \frac{p_{harmed} + p_{doomed}}{p_{helped} + p_{doomed}},$$

as shown in Appendices S4 and S6.

However, at time 2, the discrete hazard can be rewritten as

$$
\begin{aligned}
E[\Delta Y_2|\Delta Y_1 = 0, A = a] &= E[\Delta Y_2|\Delta Y_1 = 0, A = a, E_1 = 1]P(E_1 = 1|\Delta Y_1 = 0, A = a) \\
&+ E[\Delta Y_2|\Delta Y_1 = 0, A = a, E_1 = 0]P(E_1 = 0|\Delta Y_1 = 0, A = a) \\
&\overset{\text{Assumption 12--14}}{=} E[\Delta Y_2^a|\Delta Y_1^a = 0, E_1^a = 1]P(E_1^a = 1|\Delta Y_1^a = 0) \\
&+ E[\Delta Y_2^a|\Delta Y_1^a = 0, E_1^a = 0]P(E_1^a = 0|\Delta Y_1^a = 0) \\
&\overset{\text{Assumption 1}}{=} E[\Delta Y_2^a|\Delta Y_1^a = 0, E_1^a = 1, E_2^a = 1] \\
&\times P(E_2^a = 1|\Delta Y_1^a = 0, E_1^a = 1)P(E_1^a = 1|\Delta Y_1^a = 0) \\
&+ E[\Delta Y_2^a|\Delta Y_1^a = 0, E_1^a = 0, E_2^a = 1] \\
&\times P(E_2^a = 1|\Delta Y_1^a = 0, E_1^a = 0)P(E_1^a = 0|\Delta Y_1^a = 0) \\
&\overset{\text{Assumption 12}}{=} E[\Delta Y_2^{a,e_1=1,e_2=1}|\Delta Y_1^{a,e_1=1} = 0, E_1^a = 1, E_2^{a,e_1=1} = 1] \\
&\times P(E_2^a = 1|\Delta Y_1^a = 0, E_1^a = 1)P(E_1^a = 1|\Delta Y_1^a = 0) \\
&+ E[\Delta Y_2^{a,e_1=0,e_2=1}|\Delta Y_1^{a,e_1=0} = 0, E_1^a = 0, E_2^{a,e_1=0} = 1] \\
&\times P(E_2^a = 1|\Delta Y_1^a = 0, E_1^a = 0)P(E_1^a = 0|\Delta Y_1^a = 0) \\
&\overset{\text{Assumption 4, 5}}{=} E[\Delta Y_2^{a,e_1=1,e_2=1}|\Delta Y_1^{a,e_1=1} = 0] \\
&\times P(E_2^a = 1|\Delta Y_1^a = 0, E_1^a = 1)P(E_1^a = 1|\Delta Y_1^a = 0) \\
&+ E[\Delta Y_2^{a,e_1=0,e_2=1}|\Delta Y_1^{a,e_1=0} = 0] \\
&\times P(E_2^a = 1|\Delta Y_1^a = 0, E_1^a = 0)P(E_1^a = 0|\Delta Y_1^a = 0).
\end{aligned}
$$

Under Assumption 2 and the null hypothesis,

$$E[\Delta Y_2^{a,e_1=1,e_2=1}|\Delta Y_1^{a,e_1=1} = 0] = 0.$$

The conditional probability of no exposure can be rewritten as

$$P(E_1^a = 0|\Delta Y_1^a = 0) = \frac{P(\Delta Y_1^a = 0|E_1^a = 0)P(E_1^a = 0)}{P(\Delta Y_1^a = 0|E_1^a = 1)P(E_1^a = 1) + P(\Delta Y_1^a = 0|E_1^a = 0)P(E_1^a = 0)}$$
$$\overset{\text{Assumption 1}}{=} \frac{P(E_1^a = 0)}{P(\Delta Y_1^a = 0|E_1^a = 1)P(E_1^a = 1) + P(E_1^a = 0)}$$
$$\overset{\text{Assumption 12, 4}}{=} \frac{P(E_1^a = 0)}{P(\Delta Y_1^{a,e_1=1} = 0)P(E_1^a = 1) + P(E_1^a = 0)}.$$

Similarly, under Assumption 1

$$E[\Delta Y_2^{a,e_1=0,e_2=1}|\Delta Y_1^{a,e_1=0} = 0] = P(\Delta Y_2^{a,e_1=0,e_2=1} = 1, \Delta Y_1^{a,e_1=0} = 0) = P(\Delta Y_2^{a,e_1=0,e_2=1} = 1),$$

where we used that $\Delta Y_1 = 1 \implies \Delta Y_2 = 0$

Therefore,

$$E[\Delta Y_2|\Delta Y_1 = 0, A = a] = P(\Delta Y_2^{a,e_1=0,e_2=1} = 1)P(E_2^a = 1|\Delta Y_1^a = 0, E_1^a = 0)$$
$$\times \frac{P(E_1^a = 0)}{P(\Delta Y_1^{a,e_1=1} = 0)P(E_1^a = 1) + P(E_1^a = 0)}.$$

Given the data-generating mechanism, in particular the fact that there is no confounding between E_1 and E_2, we have $E_2 \perp\!\!\!\perp (A, E_1)|\Delta Y_1 = 0$. Thus

$$E[\Delta Y_2|\Delta Y_1 = 0, A = a] = P(\Delta Y_2^{a,e_1=0,e_2=1} = 1)P(E_2^a = 1|\Delta Y_1^a = 0)$$
$$\times \frac{P(E_1^a = 0)}{P(\Delta Y_1^{a,e_1=1} = 0)P(E_1^a = 1) + P(E_1^a = 0)}.$$

Moreover, in the proposed data-generating mechanism,

$$P(E_2^{a=1} = 1|\Delta Y_1^{a=1} = 0) = P(E_2^{a=0} = 1|\Delta Y_1^{a=0} = 0),$$

see Equation (19). Thus, the hazard ratio at time 2, is equal to

$$HR_2 = \frac{P(\Delta Y_1^{a=1,e_1=1}=1)}{P(\Delta Y_1^{a=0,e_1=1}=1)} \frac{P(\Delta Y_1^{a=0,e_1=1}=0)P(E_1=1)+P(E_1=0)}{P(\Delta Y_1^{a=1,e_1=1}=0)P(E_1=1)+P(E_1=0)},$$

where we used Assumption 3 to cancel out $P(E_1^a = 1)$, and the fact that under the null hypothesis $P(\Delta Y_2^{a,e_1=0,e_2=1} = 1) = P(\Delta Y_1^{a,e_1=1} = 1)$. Therefore, the hazard ratio does not only depend on the probability of the outcome given exposure, but also on the probability of the time 1 exposure. Even though the principal strata did not change over time, the time 2 hazard ratio is different from the time 1 hazard ratio by a factor of $\frac{P(\Delta Y_1^{a=0,e_1=1}=0)P(E_1=1)+P(E_1=0)}{P(\Delta Y_1^{a=1,e_1=1}=0)P(E_1=1)+P(E_1=0)}$. Thus, even if the first interval exposure increases, it is not guaranteed, that the ratio of the hazard ratios will increase or decrease monotonically, without knowledge of the distribution of the principal strata. See simulation results on testing the sharp null hypothesis of no waning (Hypothesis 0) using the equality of the hazard ratios in Appendix S9 of the main document.

S9 Simulation

Consider the following data-generating mechanism

$$\begin{aligned}
A &:= \text{Bernoulli}(0.5) \\
T_1 &:= \text{Categorical}(p_{doomed_1}, p_{helped_1}, p_{harmed_1}, p_{immune_1}) \\
E_1 &:= \text{Bernoulli}(p_{E_1}) \\
\Delta Y_1 &:= E_1 \times \big(I(T_1=1) + (1-A) \times I(T_1=2) + A \times I(T_1=3)\big) \\
E_2 &:= (1-\Delta Y_1) \times \text{Bernoulli}(p_{E_2}) \\
\Delta Y_2 &:= E_2 \times \big(I(T_2=1) + (1-A) \times I(T_2=2) + A \times I(T_2=3)\big).
\end{aligned}$$

Here, T_1 corresponds to the 4 standard principal strata, as discussed in Appendix S4. While the proof and the assumptions of Proposition 1 do not rely on the notion of the principal strata, to simulate the observed outcomes while respecting the constraints imposed on the challenge effects commonly involves assigning to all individuals their potential outcomes under all treatment levels and realizing their observed data by simulating the observed treatment. Furthermore, T_2 is determined in a way to decrease the first interval challenge effect $VE_1^{\text{challenge}} = 1 - \frac{p_{doomed_1}+p_{harmed_1}}{p_{doomed_1}+p_{helped_1}}$ such that we randomly resample a proportion of the helped individuals as doomed, to satisfy the assumption of sharp no waning of the placebo. The proportion is calculated, such that it is ensured in expectation that $\frac{p_{doomed_1}+p_{harmed_1}}{p_{doomed_1}+p_{helped_1}} \times w = \frac{p_{doomed_2}+p_{harmed_2}}{p_{doomed_2}+p_{helped_2}}$ for some factor $w > 1$, (with $w = 1$ corresponding to the sharp null hypothesis) (see Appendix S9.2 for details). $T = 1, 2, 3, 4$ are defined to correspond to being *doomed*, *helped*, *harmed*, and *immune*, respectively.

By Proposition 1, the two incidence ratios are equal when the sharp null hypothesis holds. Therefore, we can test whether $IR_1/IR_2 = 1$ to assess the null hypothesis.

We propose three alternative methods to construct the confidence interval for the fraction of the incidence ratios. We can approximate the four empirical means, corresponding to the expected outcome at the first and the second time under treatment vs no treatment, respectively, as log-normal distributions. We can calculate the variance of both IR_1 and IR_2 using the δ-method; however, the incidence ratios are negatively correlated, which depends on the level of exposure and the distribution of the susceptibility. To have a valid confidence interval, the correlation can be lower bounded by -1, but this is a considerable over-approximation when exposure is low or moderate, and when the population is heterogeneous in terms of susceptibility. To provide more precise confidence intervals, we can use

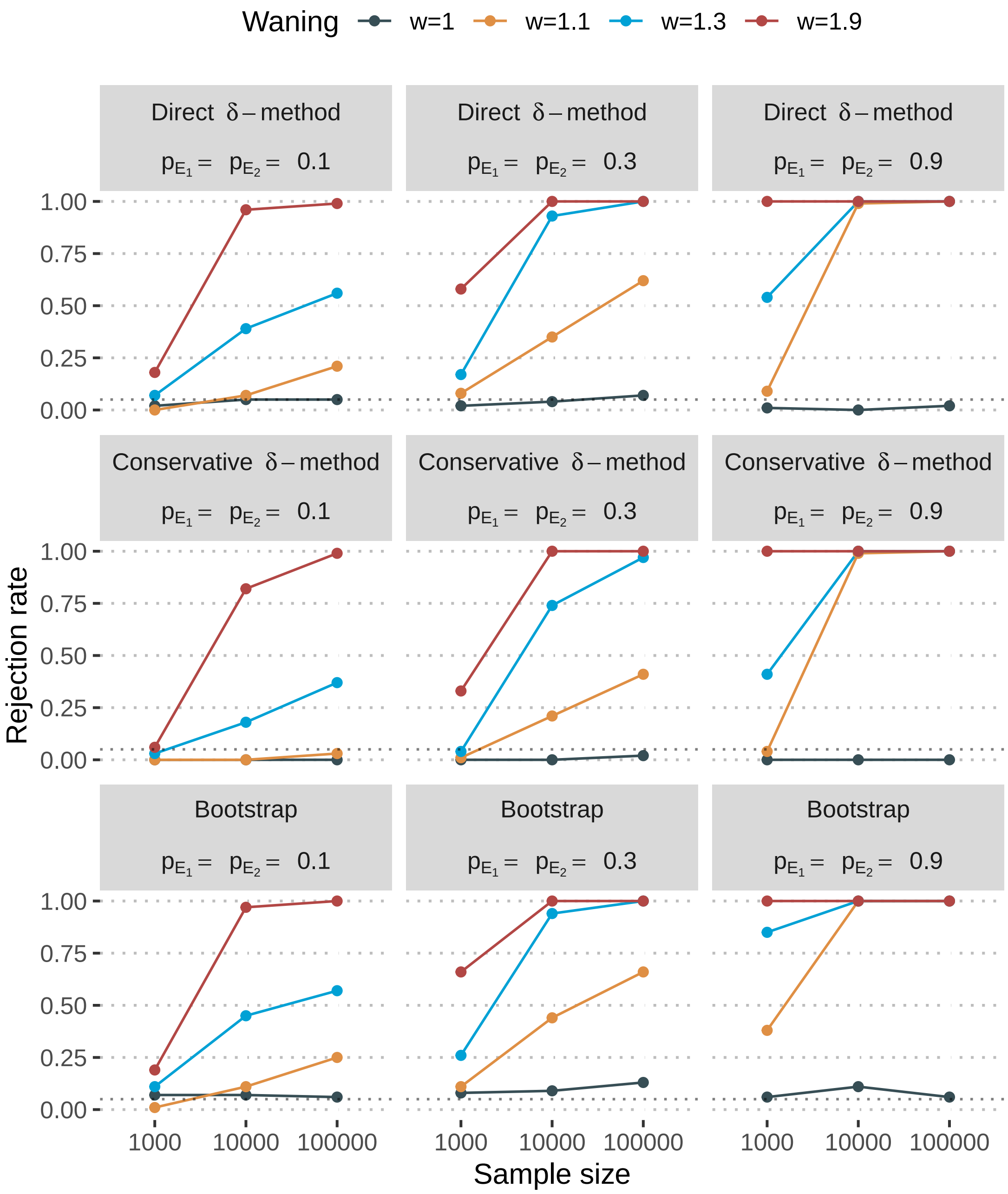

Figure S3: Rejection rate of the test statistics at level $\alpha = 0.05$ from 100 simulations. The rows correspond to the direct δ-method, the conservative δ-method, and the non-parametric bootstrap, respectively.

the δ-method to derive a direct estimator for the variance of $\log(\widehat{IR_1}) - \log(\widehat{IR_2})$. Details of both are provided in Appendix S10. Finally, given that we have access to patient-level

data, we can derive the bounds using non-parametric bootstrap methods [11].

Figure S3 illustrates the performance of the derived test statistics using the three alternatives. We set the baseline vaccine effect to $VE_1^{\text{challenge}} = 0.5$, by simulating T_1 from Categorical$(0.2, 0.6, 0.2, 0)$. Under the varying level of exposure $E_1 = E_2 \in \{0.1, 0.3, 0.9\}$, the level of overestimation of the variance is changing as illustrated in the first row. In particular, for relatively low exposure, $E_1 = E_2 = 0.1$, the test is more conservative, and for low sample size, the null hypothesis is rejected at the level of the test, even when waning is substantial, that is, the vaccine effect decreases to $VE = 0.05$. However, when the correlation approaches the supposed lower bound, by $E_1 = E_2 = 0.9$, the null hypothesis is rejected at a rate of 0.54, even for a moderate sample size of $n = 1000$, and limited waning, $VE_1 = 0.5$, $VE_2 = 0.35$. In any of the simulated scenarios, when the null hypothesis holds, the test rejects at a rate below its level.

Deriving the confidence interval using non-parametric bootstrap has greater power than using the direct δ-method. However, the δ-method maintains the nominal level, while the bootstrap shows deviations from it.

Extension to the time-to-event framework is trivial, except that the waning cannot be entirely continuous; the transition from one's reaction to exposure to a different reaction can be achieved in a discrete manner. Similar to the original paper of Janvin and Stensrud [1], we can estimate the event rates using the cumulative hazard. Whether exposure is recurring or a single one does not matter, since under the null hypothesis, the outcome will be deterministically the same following each exposure.

As discussed in Section 3, the equality of the hazard ratios is unsuitable for testing the waning of the vaccine effect, informally, due to the depletion of susceptibles over time.

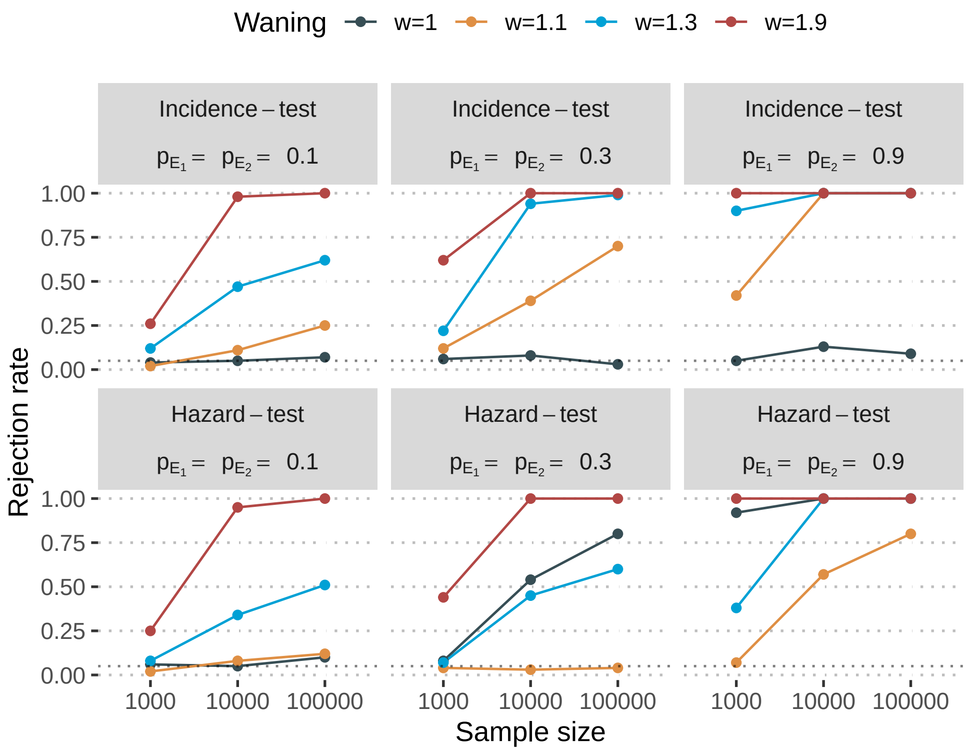

Figure S4: Rejection rates of the test statistics for $\widehat{HR}_1/\widehat{HR}_2$ at level $\alpha = 0.05$, compared to IR based test statistics, from 100 simulations, using non-parametric bootstrap.

Using the same simulated data, we constructed confidence intervals for $\widehat{HR}_1/\widehat{HR}_2$ using non-parametric bootstrap, to test $HR_1/HR_2 = 1$.

Figure S4 shows that when exposure is low, and hence the depletion of susceptibles is minor, the test based on hazard ratios is comparable to the test using incidence ratios, see Subfigure S3 C. However, when the level of infectious pressure increases by exposure, when the null hypothesis holds ($w = 0$), the test does not hold its nominal level. In addition, the power of the test does not increase with the level of the waning, which arguably is a desirable property of any sensible test of waning.

S9.1 Sensitivity analysis

Consider the same data-generating mechanism as in the main part of Appendix S9, except for T_1 and T_2. As opposed to the simulation shown in the main part of the manuscript, suppose

$$T_1 \sim \text{Categorical}(0.2, 0.4, 0.1, 0.3).$$

Under the assumption of sharp no waning of the placebo, we will consider three different scenarios for determining T_2, when the individual-level vaccine effect decreases:

- Transitioning a proportion of helped-to-doomed ($w^*_{immune} = 0$), which corresponds to the setting depicted in Figure S3, however, under a different distribution of T_1.
- Transitioning a proportion of immune-to-harmed ($w^*_{helped} = 0$).
- Transitioning an equal proportion of helped and immune to doomed and harmed, respectively ($w^*_{helped} = w^*_{immune}$).

For details of determining the exact proportions w^* to match the desired amount of waning w, see Appendix S9.2.

The simulation results are shown in Figures S5, S6, and S7.

When we include immune individuals in our cohort, by lowering the incidence rates of the outcomes, the variance of the estimator increases.

Comparing Figure S5 to S6, we can see that restricting the waning to helped-to-doomed,

Finally, even though we allow for unmeasured confounding between the exposures over time, for simplicity, we omitted that from the simulations. Consider the modified data-

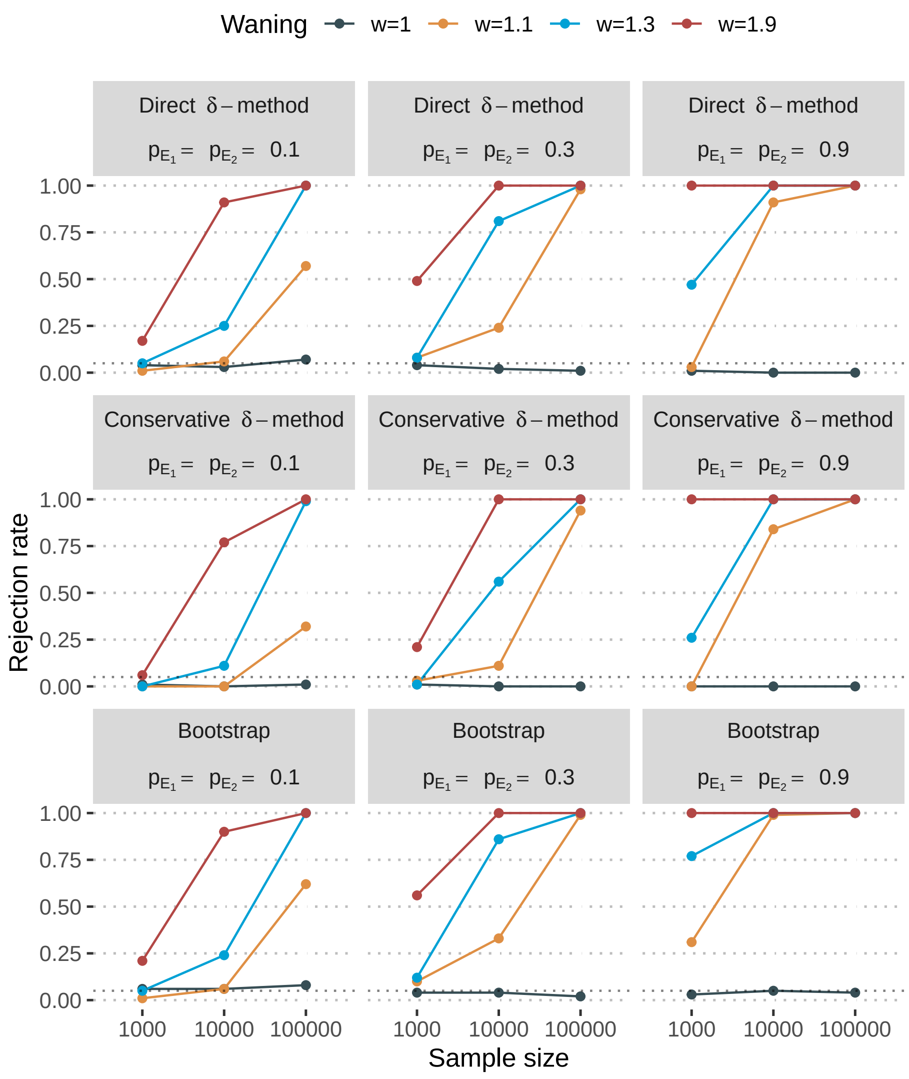

Figure S5: Rejection rates under helped-to-doomed transitions.

generating mechanism

$$A := \text{Bernoulli}(0.5)$$

$$U_E := \text{Bernoulli}(0.5)$$

$$T_1 := \text{Categorical}(p_{doomed_1}, p_{helped_1}, p_{harmed_1}, p_{immune_1})$$

$$E_1 := U_E \times \text{Bernoulli}(p_{E_{high}}) + (1 - U_E) \times \text{Bernoulli}(p_{E_{low}})$$

$$\Delta Y_1 := E_1 \times \big(I(T_1 = 1) + (1 - A) \times I(T_1 = 2) + A \times I(T_1 = 3)\big)$$

$$E_2 := (1 - \Delta Y_1) \times \big(U_E \times \text{Bernoulli}(p_{E_{high}}) + (1 - U_E) \times \text{Bernoulli}(p_{E_{low}})\big)$$

$$\Delta Y_2 := E_2 \times \big(I(T_2 = 1) + (1 - A) \times I(T_2 = 2) + A \times I(T_2 = 3)\big)$$

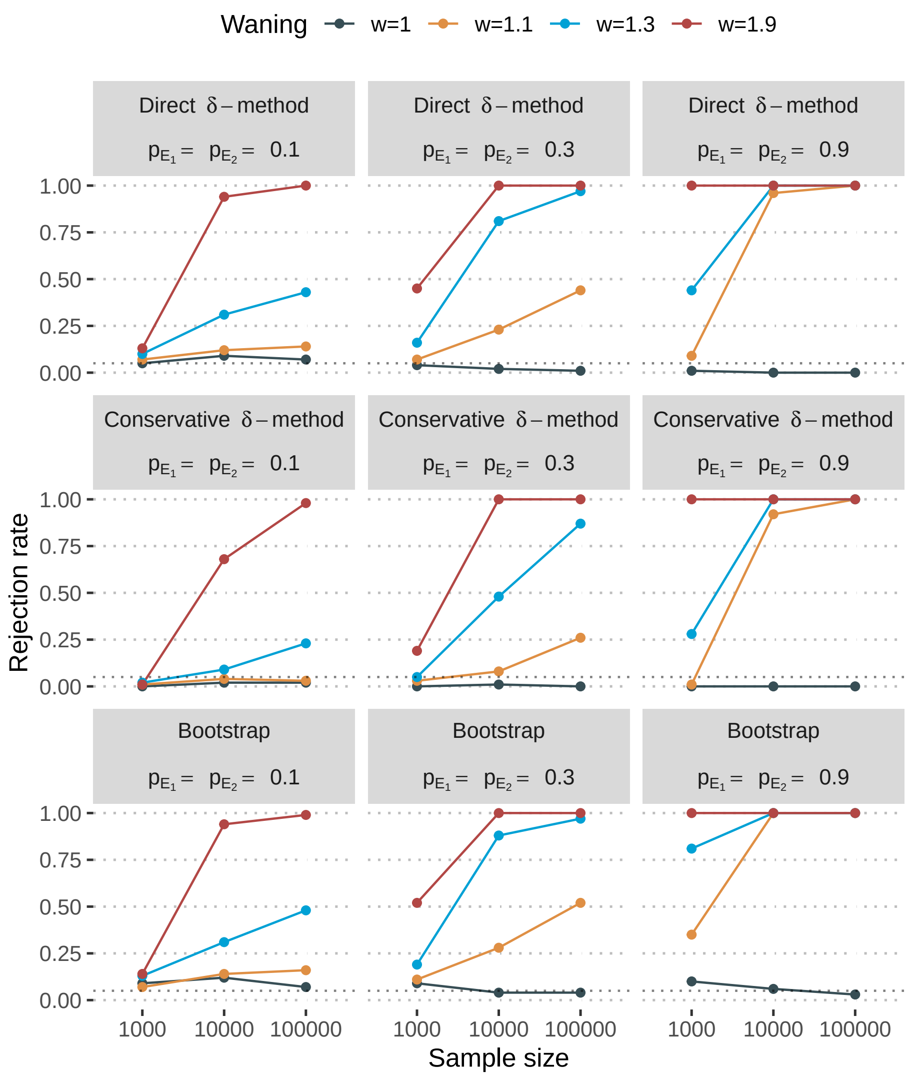

Figure S6: Rejection rates under immune-to-harmed transitions.

The binary confounder assigns each individual to exposure-seeking or exposure-averse behaviors. However, our test is valid, even when such confounding exists, as shown in Figure S8. T_1 is distributed as in the main analysis, and waning happens only through helped-to-doomed transitions. We fix $p_{E_{low}} = 0.1$ and vary $p_{E_{high}} \in \{0.1, 0.3, 0.9\}$.

In all four modified scenarios, the power gain by using the direct δ-method instead of the conservative approach is pronounced. The direct δ-method has rejection rates approximately at the level of the test statistics, while in the case of the non-parametric bootstrap,

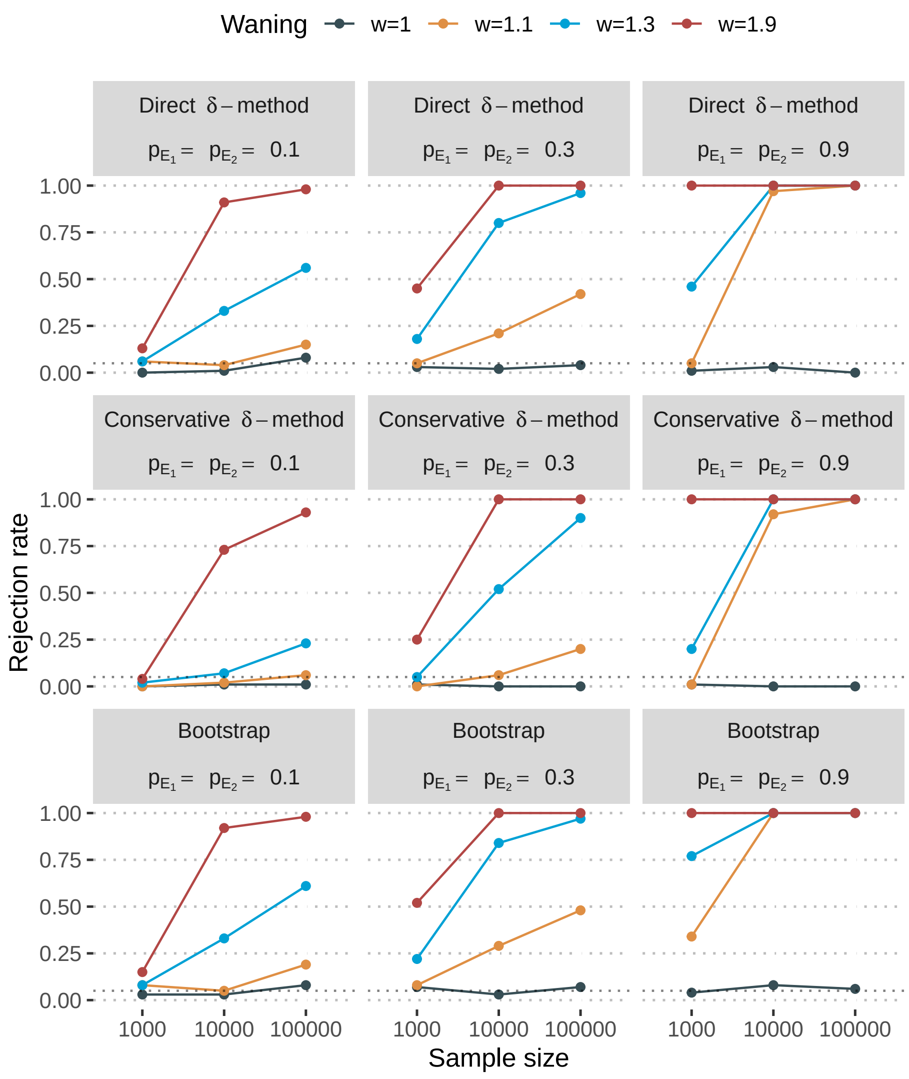

Figure S7: Rejection rates under equal helped-to-doomed and immune-to-harmed transitions.

larger deviations can happen. In general, we argue for the usage of the direct δ-method, since it is feasible using summary data only, and has acceptable power, while maintaining the validity of the test.

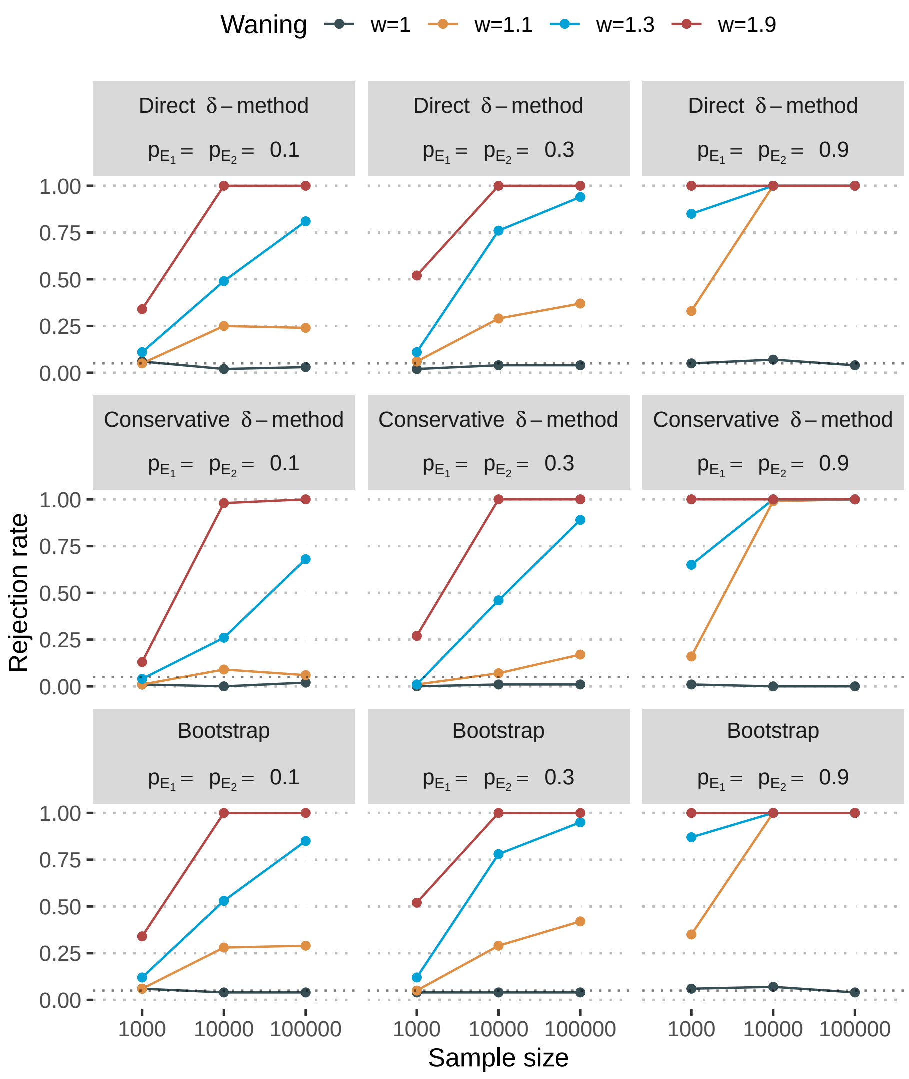

Figure S8: Rejection rates with confounded exposures.

S9.2 Determining transition rates

In Appendix S9 we simulate data, such that the distribution of the principal strata changes from time 1 to time 2. In particular, the "challenge incidence ratio" should increase, that is, the incidence ratio should increase by a factor w from time 1 to time 2 if we compare a population exposed at time 1 to a population that has been isolated first and then exposed at time 2. Consequently, the change in the principal strata, should be independent of all other variables.

We argued in Section 3, that the sharp null hypothesis is equivalent to the population level null hypothesis, under a set of conditions, one of which is sharp no waning of the placebo. That is, the individual level potential outcome will not change from time 1 to time 2 when intervening on treatment and exposure. Therefore, the only possible transitions are from "doomed" to "helped", "helped" to "doomed", "harmed" to "immune", and "immune" to "harmed". The waning of the vaccine, that is, the decrease in efficacy, corresponds to transitions from helped to doomed and immune to harmed (although, if for some, the vaccine efficacy increased, but for a larger proportion of the population it decreased, the vaccine still waned at a population level). For simplicity, in Appendix S9 we only illustrated the results for the transition helped to doomed. Simulation results with alternative waning are available in Appendix S9.1.

The incidence ratios in the challenge sense are

$$IR_1^{\text{challenge}} = \frac{p_{doomed_1} + p_{harmed_1}}{p_{doomed_1} + p_{helped_1}} \qquad IR_2^{\text{challenge}} = \frac{p_{doomed_2} + p_{harmed_2}}{p_{doomed_2} + p_{helped_2}}.$$

Suppose that the incidence rate increased by a factor of w between the two time-points, due to the transitions helped to doomed and immune to harmed. In particular, suppose that a proportion w^*_{helped} of the helped transitioned to doomed, a proportion w^*_{immune} of the immune transitioned to harmed. Then the time 2 incidence ratio can be rewritten as

$$\begin{aligned} IR_2^{\text{challenge}} &= w \times \frac{p_{doomed_1} + p_{harmed_1}}{p_{doomed_1} + p_{helped_1}} \\ &= \frac{p_{doomed_1} + p_{harmed_1} + w^*_{helped} \times p_{helped_1} + w^*_{immune} \times p_{immune_1}}{p_{doomed_1} + p_{helped_1}}. \end{aligned}$$

Solving for w^*_{helped}, w^*_{immune}, we have

$$w^*_{helped} \times p_{helped_1} + w^*_{immune} \times p_{immune_1} = (w-1) \times (p_{doomed_1} + p_{harmed_1}). \qquad (21)$$

Thus, when the only transition happening is helped to doomed,

$$w^*_{helped} = (w-1)\frac{p_{doomed_1} + p_{harmed_1}}{p_{helped_1}},$$

and when the only transition is from immune to harmed,

$$w^*_{immune} = (w-1)\frac{p_{doomed_1} + p_{harmed_1}}{p_{immune_1}}.$$

From Equation (21) it is clear, that when $w = 1$, that is, the sharp null hypothesis holds, there is no transition from either of the two possible principal strata.

S10 Variance estimation

To construct valid statistical tests for $\frac{\widehat{IR}_1}{\widehat{IR}_2}$, the limiting distribution of its estimator must be known.

Denote with $\Delta Y_i \in \{0, 1, 2\}$ as the multivariate outcome, corresponding to the outcome of interest, not happening, happening at time 1, or time 2, respectively, and denote with $A_i \in \{0, 1\}$ the treatment assignment of individual $i \in \{1, \ldots, n\}$. Then, the estimators can be defined as

$$\widehat{IR}_k := \frac{\left(\sum_{i=1}^n I(A_i = 1)I(\Delta Y_i = k)\right) / \sum_{i=1}^n I(A_i = 1)}{\left(\sum_{i=1}^n I(A_i = 0)I(\Delta Y_i = k)\right) / \sum_{i=1}^n I(A_i = 0)}$$

Using the log-normal approximation of the empirical means [12] the two incidence ratios can be approximated with

$$\log(\widehat{IR}_1) \sim \mathcal{N}(\log(IR_1), \sigma^2_{IR_1}) \qquad \log(\widehat{IR}_2) \sim \mathcal{N}(\log(IR_2), \sigma^2_{IR_2}),$$

with estimated variance $\widehat{\sigma^2}_{IR_k} = \frac{1-\sum_{i=1}^n I(A_i=1)I(\Delta Y_i=k)/\sum_{i=1}^n I(A=1)}{\sum_{i=1}^n I(A_i=1)I(\Delta Y_i=k)} + \frac{1-\sum_{i=1}^n I(A_i=0)I(\Delta Y_i=k)/\sum_{i=1}^n I(A=0)}{\sum_{i=1}^n I(A_i=0)I(\Delta Y_i=k)}$, based on the δ-method [13], and using the fact that the outcomes in the two arms are independent of each other (due to the assumption of no interference).

However, if we wish to derive the variance for $\widehat{IR}_1/\widehat{IR}_2$, or by the log-normal approximation, the variance of $\log(\widehat{IR}_1) - \log(\widehat{IR}_2)$, the outcomes of interest across time are no longer independent, thus the estimator

$$\begin{aligned}
\widehat{\sigma^2}^*_{IR_1/IR_2} &:= \widehat{\sigma^2}_{IR_1} + \widehat{\sigma^2}_{IR_2} \\
&= \frac{1 - \sum_{i=1}^n I(A_i = 1)I(\Delta Y_i = 1)/\sum_{i=1}^n I(A = 1)}{\sum_{i=1}^n I(A_i = 1)I(\Delta Y_i = 1)} + \frac{1 - \sum_{i=1}^n I(A_i = 0)I(\Delta Y_i = 1)/\sum_{i=1}^n I(A = 0)}{\sum_{i=1}^n I(A_i = 0)I(\Delta Y_i = 1)} \\
&+ \frac{1 - \sum_{i=1}^n I(A_i = 1)I(\Delta Y_i = 2)/\sum_{i=1}^n I(A = 1)}{\sum_{i=1}^n I(A_i = 1)I(\Delta Y_i = 2)} + \frac{1 - \sum_{i=1}^n I(A_i = 0)I(\Delta Y_i = 2)/\sum_{i=1}^n I(A = 0)}{\sum_{i=1}^n I(A_i = 0)I(\Delta Y_i = 2)}
\end{aligned}$$

for the variance of the difference of the log incidence ratios is biased.

Since the correlation between the incidence ratios is unknown, which we could derive only when we have access to the distribution of the principal strata and the distribution of the exposures over the two time-points, the estimated variance of the log-difference can be upper-bounded by

$$\widehat{\sigma^2}^{\text{wide}}_{IR_1/IR_2} = \widehat{\sigma^2}_{IR_1} + \widehat{\sigma^2}_{IR_2} + 2\sqrt{\widehat{\sigma^2}_{IR_1}}\sqrt{\widehat{\sigma^2}_{IR_2}}.$$

Alternatively, instead of applying the δ-method separately to IR_1 and IR_2, we can directly apply it to the ratio IR_1/IR_2.

Denote the total number no events, events at time 1, events at time 2 with $\mathbf{M}^a = \big(M_0^a, M_1^a, M_2^a\big)$ under treatment a, and the total number of individuals randomized to treatment a with N^a. Then M^a has a multinomial distribution

$$\mathbf{M}^a \sim \text{Multinomial}\big(N^a, (p_0^a, p_1^a, p_2^a)\big),$$

where p_k^a-s are determined by the distribution of the principal strata and the exposures.

The estimator of interest is

$$\widehat{IR_1/IR_2} := \frac{\sum_{i=1}^n I(A_i = 1)I(\Delta Y_i = 1)}{\sum_{i=1}^n I(A_i = 0)I(\Delta Y_i = 1)} \Bigg/ \frac{\sum_{i=1}^n I(A_i = 1)I(\Delta Y_i = 2)}{\sum_{i=1}^n I(A_i = 0)I(\Delta Y_i = 2)}$$

Using the fact that

$$\mathbf{M}^a = \Big(\sum_{i=1}^n I(A_i = a)I(\Delta Y_i = 0), \sum_{i=1}^n I(A_i = a)I(\Delta Y_i = 1), \sum_{i=1}^n I(A_i = a)I(\Delta Y_i = 2)\Big)$$

and that two arms are uncorrelated, the mean and the covariance of the estimators of interest,

$$\begin{aligned}&\mathbf{C}\\ &= \Big(\sum_{i=1}^n I(A_i = 1)I(\Delta Y_i = 1), \sum_{i=1}^n I(A_i = 1)I(\Delta Y_i = 2), \sum_{i=1}^n I(A_i = 0)I(\Delta Y_i = 1), \sum_{i=1}^n I(A_i = 0)I(\Delta Y_i = 2)\Big)^\top\end{aligned}$$

are

$$\mu := E[\mathbf{C}] = \begin{pmatrix} N_1 p_1^1 \\ N_1 p_2^1 \\ N_0 p_1^0 \\ N_0 p_2^0 \end{pmatrix} \quad \Sigma(\mathbf{C}) = \begin{pmatrix} N_1 p_1^1(1-p_1^1) & -N_1 p_1^1 p_2^1 & 0 & 0 \\ -N_1 p_1^1 p_2^1 & N_1 p_2^1(1-p_2^1) & 0 & 0 \\ 0 & 0 & N_0 p_1^0(1-p_1^0) & -N_0 p_1^0 p_2^0 \\ 0 & 0 & -N_0 p_1^0 p_2^0 & N_0 p_2^0(1-p_2^0) \end{pmatrix}$$

Using the multivariate δ-method for some $g(U_1, U_2, U_3, U_4) = \log(U_1) - \log(U_2) - \log(U_3) + \log(U_4)$ with the gradient $\nabla g(\mathbf{U}) = \Big(\frac{1}{U_1}, -\frac{1}{U_2}, -\frac{1}{U_3}, \frac{1}{U_4}\Big)^\top$, we have that the variance of $g(\mathbf{C})$ is equal to

$$\mathrm{Var}(g(\mathbf{C})) = \nabla g(\mathbf{U})^\top\big|_{\mathbf{U}=\mu}\Sigma(\mathbf{C})\nabla g(\mathbf{U})\big|_{\mathbf{U}=\mu} = \frac{1}{N_1 p_1^1} + \frac{1}{N_1 p_1^2} + \frac{1}{N_0 p_1^0} + \frac{1}{N_0 p_1^0},$$

which we can estimate by

$$\begin{aligned}\widehat{\mathrm{Var}(g(\mathbf{C}))} &= \frac{1}{\sum_{i=1}^n I(A_i = 1)I(\Delta Y_i = 1)} + \frac{1}{\sum_{i=1}^n I(A_i = 1)I(\Delta Y_i = 2)} \\ &+ \frac{1}{\sum_{i=1}^n I(A_i = 0)I(\Delta Y_i = 1)} + \frac{1}{\sum_{i=1}^n I(A_i = 0)I(\Delta Y_i = 2)}\end{aligned}$$

The derivation of the variance shows that using incidence ratios instead of hazard ratios not only has the benefit of stability when the null hypothesis holds, but also has statistical

advantages. Since the hazard ratio is calculated across different populations over time, the two arms cannot be modeled as two multinomial distributions. Their correlation is not known, as it depends on the principal strata and the exposure of individuals; therefore, to derive valid statistical tests, we have to rely on non-parametric bootstrap. Thus, having patient-level data is necessary to assess the equality of hazard ratios over time, while for the incidence ratios, summary data is sufficient.

When we only have access to summary data, such as the analysis of the BNT162b2 vaccine against COVID-19, we follow the Estimation procedure discussed by Janvin and Stensrud [1] in their Appendix.

References

[1] Matias Janvin and Mats J. Stensrud. Quantification of Vaccine Waning as a Challenge Effect. *Journal of the American Statistical Association*, 0(0):1–11, December 2024. ISSN 0162-1459. doi:10.1080/01621459.2024.2408776.

[2] M. Lipsitch, E. Goldstein, G. T. Ray, and B. Fireman. Depletion-of-susceptibles bias in influenza vaccine waning studies: how to ensure robust results. *Epidemiology and Infection*, 147:e306, 2019. ISSN 0950-2688. doi:10.1017/S0950268819001961. URL https://pmc.ncbi.nlm.nih.gov/articles/PMC7003633/.

[3] G Thomas Ray, Ned Lewis, Nicola P Klein, Matthew F Daley, Marc Lipsitch, and Bruce Fireman. Depletion-of-susceptibles Bias in Analyses of Intra-season Waning of Influenza Vaccine Effectiveness. *Clinical Infectious Diseases: An Official Publication of the Infectious Diseases Society of America*, 70(7):1484–1486, April 2020.

ISSN 1058-4838. doi:10.1093/cid/ciz706. URL https://pmc.ncbi.nlm.nih.gov/articles/PMC7318775/.

[4] Ethan Ashby, Dean Follmann, Holly Janes, Peter B. Gilbert, Ting Ye, Lindsey R. Baden, Hana M. El Sahly, and Bo Zhang. Debiasing hazard-based, time-varying vaccine effects using vaccine-irrelevant infections: An observational extension of a pivotal Phase 3 COVID-19 vaccine efficacy trial, November 2025. URL http://arxiv.org/abs/2511.15099. arXiv:2511.15099 [stat].

[5] Stephen J. Thomas, Edson D. Moreira, Nicholas Kitchin, Judith Absalon, Alejandra Gurtman, Stephen Lockhart, John L. Perez, Gonzalo Pérez Marc, Fernando P. Polack, Cristiano Zerbini, Ruth Bailey, Kena A. Swanson, Xia Xu, Satrajit Roychoudhury, Kenneth Koury, Salim Bouguermouh, Warren V. Kalina, David Cooper, Robert W. Frenck, Laura L. Hammitt, Özlem Türeci, Haylene Nell, Axel Schaefer, Serhat Ünal, Qi Yang, Paul Liberator, Dina B. Tresnan, Susan Mather, Philip R. Dormitzer, Uğur Şahin, William C. Gruber, and Kathrin U. Jansen. Safety and Efficacy of the BNT162b2 mRNA Covid-19 Vaccine through 6 Months. *New England Journal of Medicine*, 385(19):1761–1773, November 2021. ISSN 0028-4793. doi:10.1056/NEJMoa2110345.

[6] R. J. SIMES. An improved Bonferroni procedure for multiple tests of significance. *Biometrika*, 73(3):751–754, December 1986. ISSN 0006-3444. doi:10.1093/biomet/73.3.751. URL https://doi.org/10.1093/biomet/73.3.751.

[7] Yoav Benjamini and Yosef Hochberg. Controlling the False Discovery Rate: A Practical and Powerful Approach to Multiple Testing. *Journal of the Royal Statisti-

cal Society. Series B (Methodological), 57(1):289–300, 1995. ISSN 0035-9246. URL https://www.jstor.org/stable/2346101.

[8] M Elizabeth Halloran and Claudio J Struchiner. Thirty-five years of leaky vaccines. *American Journal of Epidemiology*, 194(4):918–920, October 2024. ISSN 0002-9262. doi:10.1093/aje/kwae379.

[9] Gellért Perényi and Mats Stensrud. Variant specific treatment effects with applications in vaccine studies. *Biometrics*, 81(2):ujaf068, April 2025. ISSN 1541-0420. doi:10.1093/biomtc/ujaf068.

[10] Judea Pearl. *Causality*. Cambridge University Press, Cambridge, 2 edition, 2009. ISBN 978-0-521-89560-6. doi:10.1017/CBO9780511803161. URL https://www.cambridge.org/core/books/causality/B0046844FAE10CBF274D4ACBDAEB5F5B.

[11] A. C. Davison and D. V. Hinkley. *Bootstrap Methods and their Application*. Cambridge Series in Statistical and Probabilistic Mathematics. Cambridge University Press, Cambridge, 1997. ISBN 978-0-521-57471-6. doi:10.1017/CBO9780511802843.

[12] D. Katz, J. Baptista, S. P. Azen, and M. C. Pike. Obtaining Confidence Intervals for the Risk Ratio in Cohort Studies. *Biometrics*, 34(3):469–474, 1978. ISSN 0006-341X. doi:10.2307/2530610. URL https://www.jstor.org/stable/2530610.

[13] Larry Wasserman. Introduction. In *All of Nonparametric Statistics*, pages 1–11. Springer, New York, NY, 2006. ISBN 978-0-387-30623-0.
